# Harnessing solar power: photoautotrophy supplements the diet of a low-light dwelling sponge

**DOI:** 10.1038/s41396-022-01254-3

**Published:** 2022-06-02

**Authors:** Meggie Hudspith, Jasper M. de Goeij, Mischa Streekstra, Niklas A. Kornder, Jeremy Bougoure, Paul Guagliardo, Sara Campana, Nicole N. van der Wel, Gerard Muyzer, Laura Rix

**Affiliations:** 1 Department of Freshwater and Marine Ecology, Institute for Biodiversity and Ecosystem Dynamics, University of Amsterdam, Amsterdam, Netherlands; 2 CARMABI Foundation, Piscaderabaai z/n, Willemstad, Curaçao; 3 Department of Environmental Sciences, Wageningen University and Research, Wageningen, Netherlands; 4 Centre for Microscopy, Characterisation and Analysis, University of Western Australia, Perth, WA Australia; 5 Electron Microscopy Centre Amsterdam, Amsterdam UMC, Location Academic Medical Centre, Amsterdam, Netherlands; 6 The University of Queensland, School of Chemistry and Molecular Biosciences, Australian Centre for Ecogenomics, St Lucia, QLD 4072 Australia

**Keywords:** Stable isotope analysis, Microbial ecology, Macroecology

## Abstract

The ability of organisms to combine autotrophy and heterotrophy gives rise to one of the most successful nutritional strategies on Earth: mixotrophy. Sponges are integral members of shallow-water ecosystems and many host photosynthetic symbionts, but studies on mixotrophic sponges have focused primarily on species residing in high-light environments. Here, we quantify the contribution of photoautotrophy to the respiratory demand and total carbon diet of the sponge *Chondrilla caribensis*, which hosts symbiotic cyanobacteria and lives in low-light environments. Although the sponge is net heterotrophic at 20 m water depth, photosynthetically fixed carbon potentially provides up to 52% of the holobiont’s respiratory demand. When considering the total mixotrophic diet, photoautotrophy contributed an estimated 7% to total daily carbon uptake. Visualization of inorganic ^13^C- and ^15^N-incorporation using nanoscale secondary ion mass spectrometry (NanoSIMS) at the single-cell level confirmed that a portion of nutrients assimilated by the prokaryotic community was translocated to host cells. Photoautotrophy can thus provide an important supplemental source of carbon for sponges, even in low-light habitats. This trophic plasticity may represent a widespread strategy for net heterotrophic sponges hosting photosymbionts, enabling the host to buffer against periods of nutritional stress.

## Introduction

Mixotrophy is a widespread and important nutritional strategy in terrestrial and marine ecosystems [1], from autotrophic land plants that have acquired heterotrophic feeding modes (e.g., parasitism, carnivory) [2], to reef-building corals with photosynthetic symbionts [3], or abundant plankton in the sunlit ocean [4]. This successful strategy enables organisms to buffer against fluctuations in resource availability by modulating autotrophic or heterotrophic nutritional modes. The dynamic nature of the marine environment is thought to favour mixotrophy, where mixotrophs are broadly distributed and create important trophic linkages [5]. While key benthic groups such as scleractinian corals [1] and soft corals [6] are widely regarded as mixotrophic, our understanding of the prevalence of this feeding mode in sponges is relatively limited, despite their important role in ecosystem functioning. Mixotrophy is believed to be a widespread strategy for sponges residing in euphotic ecosystems, but the contribution of both autotrophy and heterotrophy to carbon diet and their impact on elemental cycling at the individual or ecosystem level has seldom been quantified [7, 8].

Sponges are a highly successful group of benthic invertebrates, found in abundance from deep-sea to shallow-water marine ecosystems [7]. They are among the oldest extant metazoans [9] and their successful global dispersal has been, for the major part, attributed to their efficient use of nutrients—a result of the voracious filter-feeding capacity of the host and its diverse array of symbiotic microorganisms [10, 11]. Sponges host some of the most complex and diverse communities of microorganisms of any holobiont in the marine realm [12], which help their host adapt to a wide range of environments by expanding their metabolic capabilities [13, 14]. While sponges are heterotrophic organisms, many species residing in shallow, light-fuelled ecosystems have gained the ability to photosynthesize by harbouring photosynthetic symbionts. These include dinoflagellates, diatoms, chlorophytes, and rhodophytes [15–17], but the most prevalent photosymbionts are cyanobacteria [18]. In sponges, cyanobacteria are thought to live in mutualistic symbiosis with the host, capitalizing on a nutrient rich and sheltered environment within the sponge body, while providing their host with photosynthates [19], bioavailable nitrogen (via nitrogen fixation) [20], UV protection [21], and anti-predator secondary metabolites [22].

Our understanding of the contribution of carbon fixation by cyanobacteria to sponge nutrition has typically focused on sponges that dwell in shallow, high-light environments, particularly coral reefs [23], where they can derive a significant proportion (e.g., up to 80%) of their carbon requirements from their photosymbionts [24–28]. Sponges where symbiont photosynthesis exceeds holobiont respiration are termed “net phototrophic” and are estimated to have >50% of their respiratory needs met by their photosynthetic partners [29]. However, net phototrophic sponges—which often adopt flattened foliose morphologies to maximize photosynthesis [24]—represent only a minority of cyanobacteria-bearing sponges. Many “net heterotrophic” sponges harbour cyanobacterial symbionts in their tissue [18], with cyanobacteria representing one of the most common microbial lineages across a range of sponge species globally [11, 18]. Furthermore, many sponges live in environments where light levels are low: shaded, cryptic reef habitats (e.g., crevices and the interstitial spaces of the coral reef framework) are often dominated by diverse communities of sponges [30–33]. For sponges residing in such habitats, the contribution of cyanobacteria to host metabolism has received little attention [34, 35].

Assessing the role of cyanobacterial carbon fixation to host nutrition is complicated by the fact that photosynthesis and translocation rates are not necessarily closely coupled [28]. Manipulative experiments have shown that while cyanobacteria enhance host growth in some sponge species, they do not appear to translocate nutrients to their host in others [28, 36–39]. Thus, photosynthesis and respiration measurements alone are insufficient to infer the degree of dependency of the host on cyanobacteria-derived nutrition, and additional metrics are needed to confirm metabolic interactions [28]. Stable isotope probing (SIP) provides such a tool, and has been used to trace the transfer of photosynthetically fixed carbon from dinoflagellates of the family Symbiodiniaceae or cyanobacteria to the sponge host [13, 23]. The coupling of SIP with nanoscale secondary ion mass spectrometry (NanoSIMS) has also facilitated the visualization of metabolic interactions at the cellular level in photosymbiotic sponges, for example by proving the mutualistic relationship between Symbiodiniaceae and a bioeroding sponge [40].

Here, we estimated the contribution of photosynthetic carbon fixation to the respiratory demand and total carbon diet of the coral reef sponge *Chondrilla caribensis* and assessed whether the sponge host benefits from the autotrophic assimilation of inorganic carbon and nitrogen by cyanobacteria. This common Caribbean sponge hosts a high density of symbiotic microorganisms (termed a “high microbial abundance”, or HMA, sponge [41]), including the most common sponge cyanobacterial symbiont: *Candidatus* Synechococcus spongiarum [18]. It is considered a net heterotrophic sponge [23] and is commonly found at depths of around 20 m where it predominantly resides on vertical surfaces or underneath reef projections where light levels are low. We assessed the photosynthetic response of *C. caribensis* by measuring respiration and primary productivity and quantified daily gross carbon fixation rates over a diel cycle. We then measured the natural heterotrophic carbon diet of *C. caribensis* in situ in order to quantify the relative contribution of photoautotrophy and heterotrophy to total carbon acquisition. Finally, we combined SIP and NanoSIMS to test for the transfer of photosynthates between symbiotic cyanobacteria and the sponge host at the single-cell level. Since carbon and nitrogen assimilation are tightly linked in cyanobacteria, we simultaneously visualized the incorporation of both inorganic carbon (HCO_3_^-^) and nitrogen (NH_4_^+^) by (i) cyanobacteria, (ii) other prokaryotes, and (iii) sponge cells over time, confirming the translocation of symbiont-assimilated nutrients to the host. This holistic approach enabled us to comprehensively assess the contribution of symbiotic cyanobacteria to the mixotrophic diet of a sponge residing in low-light environments.

## Materials and methods

### Sponge collection

**Fig. 1:**
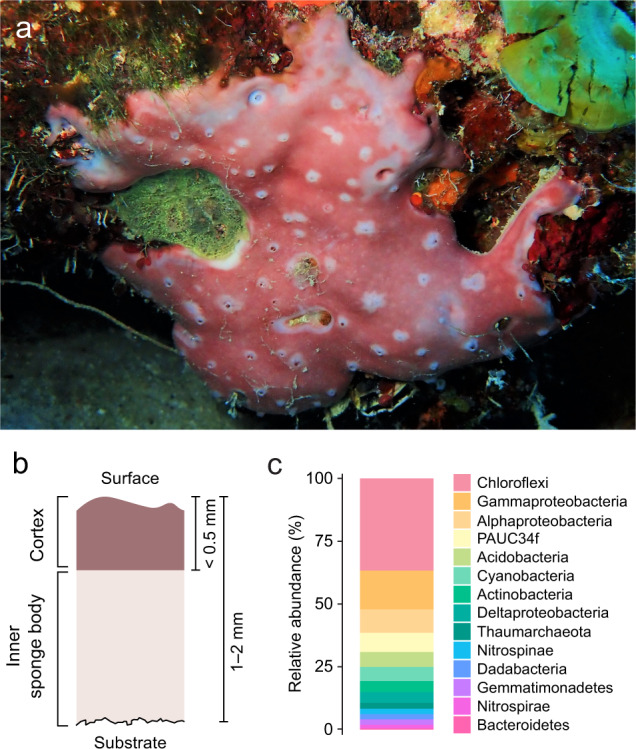
The cyanobacteria-hosting sponge *Chondrilla caribensis*. **a** In situ image at 20 m water depth. **b** Schematic diagram of the sponge tissue perpendicular to the sponge surface. The symbiotic cyanobacteria *Candidatus* Synechococcus spongiarum resides in both the collagen-rich cortex and inner sponge body. **c** Composition of the microbial community based on analysis of the 16S rRNA gene (*n* = 3). Phyla are shown, with Proteobacteria split into classes. The cyanobacterial phylum consists solely of *Ca*. Synechococcus spongiarum.

This study was conducted at the Caribbean Research and Management of Biodiversity (CARMABI) Research Station on the Southern Caribbean island of Curaçao from October to December 2019. Individuals of the sponge *Chondrilla caribensis* forma *hermatypica* [42] (Porifera, Demospongiae) were collected from the house reef in front of CARMABI (12° 07’ 14” N, 68° 58’ 12” W) between 18 and 22 m water depth using SCUBA (Fig. 1). This morphological type grows as thin encrustations (1–2 mm thick) on reef substrate and can be found commonly between 15 and 25 m, with highest abundances at around 20 m water depth. It resides primarily on vertical surfaces and under ledges, but also on exposed benthic surfaces and within caves (Supplementary Fig. 1). Sponges were chiselled from the reef, cleared of epibionts, and shaped to an approximate surface area of 13 ± 4 cm^2^ (mean ± SD throughout unless otherwise stated), with at least 5 intact oscula (outflow openings). Sponge individuals were stored vertically in sheltered wire cages on the house reef (15 m water depth) for between 1–2 weeks prior to experimentation, ensuring photoacclimation of all sponges to a light profile mimicking irradiances on vertical surfaces at around 20 m depth (Supplementary Table 1). Only healthy, pumping sponge individuals were used in the experiments. Additional specimens (*n* = 3) were collected in situ at around 20 m water depth and subsequently assessed for microbial community composition (see Supplementary Methods).

### Photosynthesis–irradiance curves

Photosynthesis–irradiance (*P*–*E*) curves were constructed for *C. caribensis* by measuring net oxygen fluxes ex situ at 7 increasing irradiance levels of natural sunlight (between 0 and ~800 μmol photons m^−2^ s^−1^) (*n* = 4). Sponge individuals were incubated in individual airtight 2-L chambers [43] filled with natural seawater. Irradiance levels were achieved by wrapping the chambers in 6 layers of cloth and enclosing each in opaque PVC tubing with a lid (i.e., irradiance of 0 μmol photons m^−2^ s^−1^, *E*_*0*_) and then sequentially removing the PVC tube and layers of cloth, from 6 layers to none (see Supplementary Methods).

Rates of net oxygen consumption or production at each irradiance level were calculated using the linear slope of oxygen concentration, and fluxes expressed as μmol O_2_ cm^−2^_sponge_ h^−1^. *P–E* curves were fitted using the exponential function [44]:

1 $$P_E = P_{max} \times \left( {1 - e^{\left( { - E/E_k} \right)}} \right) + R_d$$

where *P*_*E*_ is the hourly net photosynthetic rate at a given irradiance (*E*), *P*_*max*_ is the maximum gross photosynthetic rate, *E*_*k*_ is the saturating irradiance coefficient, and *R*_*d*_ is the dark respiration rate (measured at *E*_*0*_), which is negative as oxygen is consumed. All photokinetic parameters are given in Supplementary Table 2.

### The mixotrophic carbon diet of *C. caribensis*

A daily mixotrophic carbon diet for *C. caribensis* was constructed based on typical irradiances received on vertical reef surfaces at 20 m water depth. To achieve this, we combined the *P–E* curves and a daily ambient light profile at 20 m with in situ natural diet incubations to determine photoautotrophic and heterotrophic daily uptake rates of carbon.

Daily photoautotrophic carbon uptake rates were quantified for sponges residing on vertical surfaces. The light profile of a vertical surface at 20 m was derived from a model [45] that was adjusted to match ambient irradiances measured in situ at the house reef of CARMABI (see Supplementary Methods; Supplementary Table 1). The light profile was then integrated against the exponential function of the *P–E* curves to obtain hourly gross oxygen fluxes, which were summed to give daily gross primary productivity rates (*P*_*g*_). Daily net primary productivity rates (*P*_*n*_) were calculated as

2 $$P_n = P_g + R$$

where daily respiration (*R*) is calculated from dark respiration rates (24 x *R*_*d*_; Eq. 1). For each sponge replicate, *P*_*g*_, *P*_*n*_, and *R* rates were calculated, as well as *P*_*g*_*:R* ratios (termed daily P:R ratios). Oxygen fluxes were then converted to carbon fluxes using a photosynthetic quotient of 1.1 [46] and a respiratory quotient of 0.75 (published values range from ~0.7–0.8 [47–49]), respectively, and expressed as μmol C cm^−2^_sponge_ d^−1^. We acknowledge that the use of *R*_*d*_ to calculate *R* may have led to an underestimation of daily net primary productivity rates (*P*_*n*_), as *R*_*d*_ was measured in darkness during the day. Separate incubations conducted in darkness during the day and at night showed that there was a small decrease in respiration rates at night (−1.15 ± 0.52 versus −0.94 ± 0.50 μmol O_2_ cm^−2^_sponge_ h^−1^).

The net heterotrophic carbon diet of *C. caribensis* was measured by incubating sponge individuals in situ in both dark and light conditions (*n* = 6 per condition). Natural diet incubations were conducted over one week in December 2019, between 10:00 and 12:00 h near station “Buoy 1” (12° 07’ 28.65” N, 68° 58’ 23.23” W) at 10 m water depth. Sponges were incubated in airtight 2-L chambers and water samples (100 mL) were taken with acid-washed polypropylene syringes at *t* = 0, 5, 10, 20, and 40 min. For light incubations, chambers were wrapped in 2 layers of shade cloth to mimic maximal irradiance levels on vertical surfaces at 20 m (~49 ± 26 μmol photons m^−2^ s^−1^, measured over the course of 3 consecutive days). For dark incubations, chambers were wrapped in opaque cloth. Water samples were stored at 4 °C in the dark at the surface prior to processing within 5 h. Duplicate samples were taken for dissolved organic carbon (DOC) and live (i.e., planktonic) particulate organic carbon (LPOC) (details in Supplementary Methods). A seawater only control was run daily (*n* = 3 per light condition). At the end of the experiment, sponges were imaged for surface area analysis using Image J (http://rsb.info.nih.gov/ij/), and tissue collected and stored at −20 °C before subsequent lyophilization and dry weight measurement.

Initial net hourly DOC uptake rates by sponges were calculated by applying the previously described bi-exponential “2G model”, which describes uptake of the labile and refractory portion of DOM [50, 51]. For initial net hourly LPOC removal rates by sponges, an exponential clearance of planktonic cells in a well-mixed system was assumed [52] (see Supplementary Methods for details of water sample analyses and calculation of carbon uptake rates; Supplementary Fig. 2). Where models did not significantly fit the data, and *post hoc* linear regressions did not show significant removal or release rates, uptake rates were set to 0. Hourly fluxes of DOC and LPOC obtained in the dark and in the light were each multiplied by 12 and then summed to calculate daily net rates of DOC and LPOC uptake, respectively (DOC_24_ and LPOC_24_).

Finally, the total mixotrophic diet of *C. caribensis* was estimated by summing daily carbon uptake from photoautotrophy (*P*_*g*_) and net daily heterotrophy (DOC_24_ + LPOC_24_), expressed as μmol C cm^−2^_sponge_ d^−1^. The standard deviation of the total mixotrophic diet was obtained by propagating the standard deviations of the underlying fluxes according to:

3 $$SD_{mixotrophic\,diet} = \sqrt {{SD_{P_g}}^2 + {SD_{DOC_{24}}}^2 + {SD_{LPOC_{24}}}^2}$$

The relative contribution of autotrophic carbon uptake to the total mixotrophic carbon uptake was also calculated.

### Pulse-chase isotopic labelling experiment

To visualize photosynthetic carbon fixation and ammonium assimilation by *C. caribensis* at the cellular level and assess metabolic integration between the microbiome and host, a pulse-chase experiment was conducted using isotopically labelled artificial seawater (ASW). Sponges were incubated with ASW enriched with ^13^C sodium bicarbonate (NaH^13^CO_3_; Cambridge Isotope Laboratories, 99% ^13^C) and ^15^N ammonium chloride (^15^NH_4_Cl; Cambridge Isotope Laboratories, 99% ^15^N) to a final concentration of 2 mM and 5 µM, respectively, as per Pernice et al. [53]. Sponges were incubated in individual airtight chambers of 3 L and chamber lids were fitted with a magnetic stirring device to ensure water mixing. Chambers were partially submerged in an outdoor flow-through aquarium over a 6-h pulse period (starting at 06:00 h) under low natural-light conditions (~50 μmol photons m^−2^ s^−1^ at midday, reflecting maximal irradiances received on vertical surfaces at 20 m depth). Sponges were then transferred to flow-through aquaria with non-labelled fresh running seawater for the 42-h chase period (see Supplementary Methods). Non-labelled sponges provided background enrichment values (*t* = 0) and sponges were also incubated with enriched ASW for 6 h in the dark. Four independent sponge replicates were sampled at each time-point (*t* = 0, 6, 6 (dark), and 48 h). At each time-point, a subsample of tissue was removed with a sterile scalpel blade and fixed in 2.5% (v/v) glutaraldehyde + 1% (w/v) paraformaldehyde in PHEM buffer (1.5 x PHEM (60 mM PIPES, 25 mM HEPES, 10 mM EGTA, 2 mM MgSO_4_.7H_2_O) and 9% (w/v) sucrose, pH 7.4), and the remaining “bulk” tissue transferred to pre-weighed cryovials and stored at −20 °C for subsequent bulk tissue stable isotope analysis (see Supplementary Methods).

### Electron microscopy and NanoSIMS

**Fig. 2:**
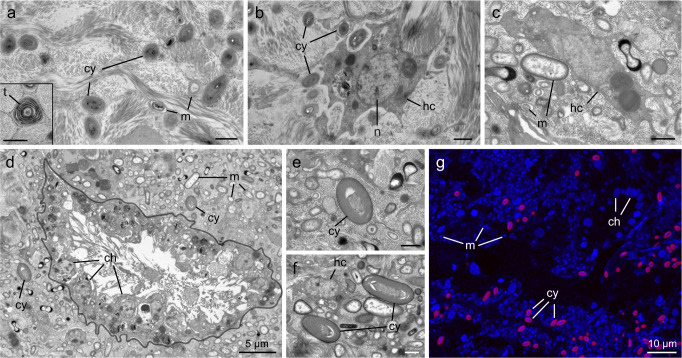
Transmission electron- and fluorescence-microscopy images showing the cellular ultrastructure of *Chondrilla caribensis*. Symbiotic cyanobacteria and other microorganisms reside in the collagen-rich cortex of the sponge (**a**, **b**) and the inner sponge body (**c**–**g**). The inner sponge body includes the extracellular mesohyl matrix and choanocyte chambers, comprised of individual host filter-cells (choanocytes). Symbiotic microorganisms dominate the mesohyl of the inner sponge body (**d**), while cyanobacteria are more prevalent in the cortex (**a**). DAPI-stained image of the inner sponge body highlighting the location of microbial symbionts and host cells (**g**). Cyanobacteria are distinguished in red by the autofluorescence of their photosynthetic pigments. ch choanocyte, cy cyanobacteria, hc host cell, m symbiotic microorganism, n nucleus, t thylakoids. The grey line in (**d**) delineates a choanocyte chamber. Scale bars are 1 µm unless otherwise stated.

Fixed tissue samples were processed and embedded as per Hudspith et al. [54]. Semithin sections (500 nm) were sectioned perpendicular to the sponge surface and transferred to silicon wafers, stained with uranyl acetate and lead citrate, and imaged with a Zeiss Sigma Field Emission Scanning Electron Microscope (SEM) at 8 kV at the Electron Microscopy Centre Amsterdam (EMCA). Regions of interest were imaged and digitally mapped for subsequent NanoSIMS analysis. Transmission electron microscope (TEM) and fluorescence images were also taken from a subset of samples (Fig. 2; see Supplementary Methods).

To visualize the assimilation of inorganic carbon and nitrogen at the single-cell level, areas imaged by SEM were analyzed using a NanoSIMS 50 ion probe (CAMECA, Paris, France) at the Centre for Microscopy, Characterisation and Analysis (University of Western Australia, Perth) following the methodology outlined by Hudspith et al. [54]. Between 4 to 8 different areas were scanned per sample, with a typical raster size of 40 × 40 µm and resolution of 512 × 512 pixels. NanoSIMS analysis focused on 3 regions of interest (ROI) in both the collagen-rich outer cortex of the sponge and the inner sponge body (Fig. 1b). Host cells (1), cyanobacteria (2), and all other symbiotic microorganisms (3; henceforth termed “symbiotic microorganisms”) were scanned (Fig. 2). Choanocytes—filter-feeding sponge cells—were excluded from analysis, as occasionally, they indirectly became enriched in ^15^N by consuming the few remaining bacteria from the ASW medium that had incorporated ^15^N ammonium (^13^C enrichment was negligible). ROI were drawn over NanoSIMS ^12^C^14^N mass images, using ^31^P and SEM images as a reference to identify cell types. Extracted isotopic ratios (*R*_*sample*_) were multiplied by a correction factor based on daily scans of *Saccharomyces cerevisiae* (yeast; see Hudspith et al. [54]) and then expressed in standard delta notation (‰):

4 $$\Delta \delta ^{13}{{{{{{{\mathrm{C}}}}}}}}\,{{{{{{{\mathrm{or}}}}}}}}\,\Delta \delta ^{15}{{{{{{{\mathrm{N}}}}}}}} = \left( {\frac{{R_{sample}}}{{R_{ref}}} - 1} \right){{{{{{{\mathrm{x}}}}}}}}\,1000$$

where *R* is the measured ^13^C/^12^C or ^15^N/^14^N ratio of the sample or unlabelled controls (*ref*). Unlabelled sponge tissue (*t* = 0) was analyzed to obtain natural abundance stable isotopic ratios for each ROI category and 2 biological replicates were analyzed per time-point (*t* = 0, 6, 6 (dark)), except for *t* = 48, where 3 replicates were analyzed. A total of 9421 cells were scanned (Supplementary Table 3). Individual cells were considered enriched if their Δδ^13^C or Δδ^15^N values were more than 3 times the standard deviation of unlabelled control ROI (Supplementary Fig. 3).

### Statistical analyses

Bulk stable isotope and extracted NanoSIMS data were analyzed in Primer v7 [55] using the add-on PERMANOVA+ [56]. Univariate and multivariate permutational analysis of variances (PERMANOVAs) were used to test for significant differences between groups for bulk tissue and NanoSIMS data, respectively. Resemblance matrices were constructed using Euclidean distances and analyses performed using Type III partial sum of squares under the reduced model (4999 permutations). Data from biological replicates were pooled per time-point for each ROI category for analysis of the NanoSIMS data. *Post hoc* pairwise comparisons were made to determine which levels of factors were significant.

*P–E* curves were fitted and graphed using SigmaPlot (v. 14.5) and the change in irradiance over the course of a day on a vertical surface integrated against the fitted *P–E* curves using R (v. 4.0.3). Models used to calculate carbon uptake rates for individual replicates were run in Excel (with Solver add-on; 2G model) and SPSS (software v 25; inverse exponential and linear regression). Differences in net carbon uptake rates between dark and light incubations were analyzed using 2-tailed paired t-tests (SPSS). Significance was determined at the *p* = 0.05 level. Full statistical outputs are available in Supplementary Tables 4 and 5.

## Results

### *Chondrilla caribensis* is net heterotrophic and adapted to low-light conditions

**Fig. 3:**
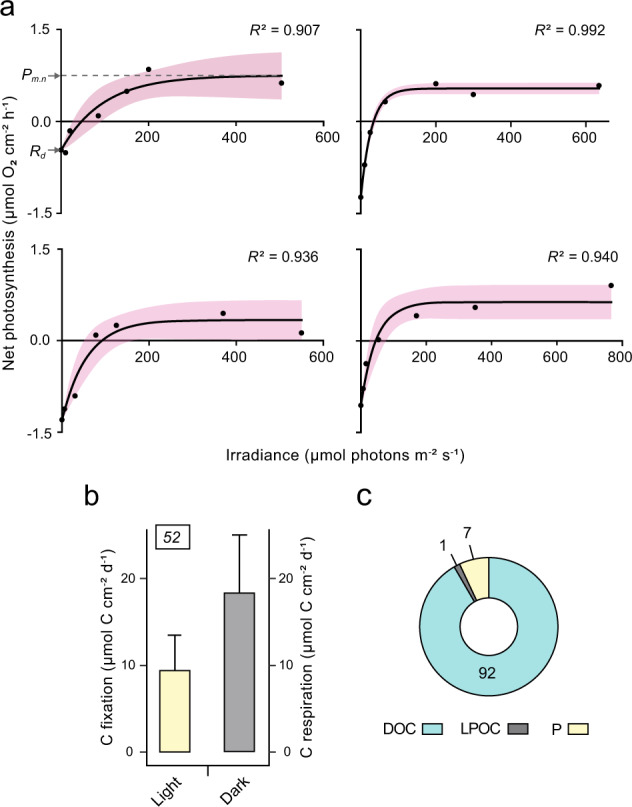
Photoautotrophic and heterotrophic nutrient uptake in *Chondrilla caribensis*. **a** Photosynthesis–irradiance (*P*–*E*) curves for 4 sponge individuals, fitted with an exponential function [44]. The shaded areas represent 95% confidence bands. The maximum net photosynthetic rate (*P*_*m.n*_) and respiration rate (*R*_*d*_) are annotated in the first curve. Note the different *x* axes. **b** Flux of daily gross photosynthetic carbon fixation at 20 m water depth and the 24 h respiratory carbon demand of the sponge (grey bar). The number above the bar shows carbon fixation as a percentage of the respiratory carbon demand. Data derived from *P*–*E* curves (*n* = 4). **c** The contribution of heterotrophy (DOC + LPOC) and photoautotrophy (P) to total daily carbon uptake. Numbers represent the percent contribution of a given fraction to total carbon uptake (*n* = 6 for DOC/LPOC data, *n* = 4 for photosynthesis data). Data in (**b**) represent mean ± SD. DOC dissolved organic carbon, LPOC live particulate organic carbon.

*Chondrilla caribensis* displayed a photosynthesis–irradiance response typical of sponges hosting photosymbionts (Fig. 3a) and the relationship between photosynthetic activity and irradiance was well characterized by an exponential model (*R*^2^ ≥ 0.907; Supplementary Table 2). The steep initial slopes of the *P–E* curves and low saturating light intensities (*E*_*k*_, 57 ± 28 µmol photons m^−2^ s^−1^) indicate an efficient use of light at low photon flux rates. The average daily ratio of gross photosynthesis to respiration (P:R), which represents an index of the energetic budget over a diel cycle, was 0.35 ± 0.08. This sponge is therefore net heterotrophic at 20 m water depth, as indicated by P:R ratios <1. Accordingly, gross primary productivity did not have the potential to balance the respiratory demand of this sponge, with daily net primary productivity being −8.8 ± 4.4 μmol C cm^−2^_sponge_ d^−1^ (−392 ± 195 μmol C g DW^−1^ d^−1^). However, carbon fixation via photosynthesis (gross primary productivity: 9.4 ± 4.1 μmol C cm^−2^_sponge_ d^−1^) was estimated to contribute to the daily respiratory demand of the holobiont (18.3 ± 6.8 µmol C cm^-2^_sponge_ d^−1^; Fig. 3b) by 52 ± 11%, which represents the maximum amount of carbon potentially available to the entire symbiotic assemblage (i.e., host, cyanobacteria, symbiotic microorganisms).

### Heterotrophic uptake exceeds photoautotrophic carbon assimilation in the diet of *C. caribensis*

Dissolved organic carbon (DOC; net uptake rates of 127 ± 63 µmol C cm^−2^_sponge_ d^−1^) dominated the heterotrophic diet of *C. caribensis* and provided 98% of the total organic carbon uptake, whereas live particulate organic carbon (LPOC) represented only 2% of the total uptake. Average removal rates of DOC were higher in the dark compared to the light, and the inverse was true for LPOC (Supplementary Fig. 2; Supplementary Table 6), but these differences were not statistically significant (DOC: *t* = 2.087, *p* = 0.091 and LPOC: *t* = −1.864, *p* = 0.121; df = 5 for both). When the contribution of photosynthetically fixed carbon was included, the daily total carbon uptake rate amounted to 139 ± 63 µmol C cm^‒2^_sponge_ d^−1^, with photoautotrophy by cyanobacteria accounting for 7 ± 3% of the total carbon uptake (Fig. 3c).

### The microbial community of a mixotrophic sponge

Populations of cyanobacterial cells were observed in the cortex and inner sponge body of *C. caribensis*, with cells displaying characteristic spiral thylakoids (Fig. 2). Cyanobacterial cells were more densely populated in the cortex and generally smaller, with an average width of 0.84 ± 0.16 μm compared to 1.28 ± 0.13 μm in the inner sponge body. Cyanobacteria became sparse deeper down into the tissue, and thus the cortex and inner sponge body were separately analyzed with NanoSIMS. 16S rRNA gene amplicon sequencing confirmed the identity of these cells as *Candidatus* Synechococcus spongiarum. The microbial community was dominated by Chloroflexi (37%), Gamma- (16%), and Alpha-proteobacteria (9%), with *Ca*. S. spongiarum accounting for 6% of the microbial community (Fig. 1c).

### Carbon fixation is driven by cyanobacteria with translocation of ^13^C to host cells

**Fig. 4:**
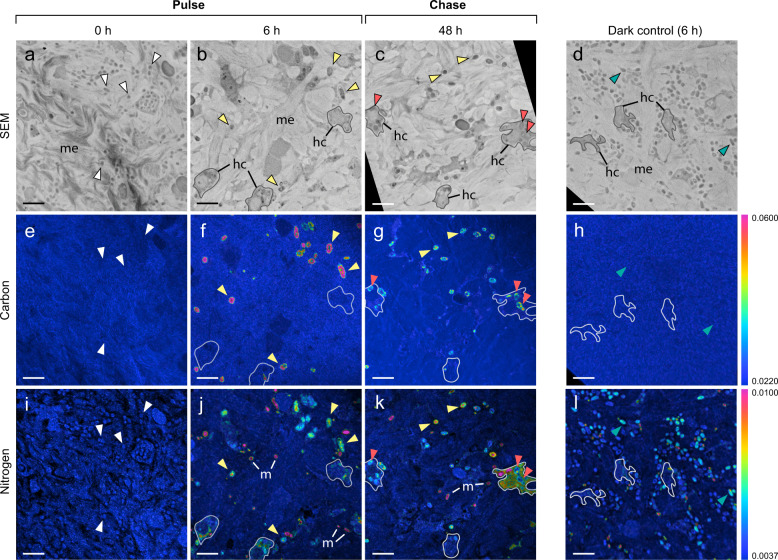
The cortex of *Chondrilla caribensis*: single-cell visualization of inorganic ^13^C-bicarbonate and ^15^N-ammonium assimilation. SEM images (**a**–**d)** show the location of resident symbiotic cyanobacteria, microorganisms, and host cells. Corresponding NanoSIMS images show the distribution of ^13^C/^12^C (**e**–**h**) and ^15^N/^14^N (**i**–**l**) at the end of the pulse (6 h) and chase period (48 h) in relation to non-labelled tissue (0 h) and the dark control. The colour scale represents enrichment relative to natural abundance ratios (in blue, 2 × 0.011 for ^13^C/^12^C and 0.0037 for ^15^N/^14^N). Arrows depict cyanobacteria: white for unlabelled cells (0 h), yellow for cyanobacteria highly enriched in ^13^C and ^15^N, red for enriched cyanobacteria engulfed by host cells, and green for ^15^N-enriched cyanobacteria of the dark control. hc host cell, m symbiotic microorganism, me mesohyl. Scale bars are 5 µm.

**Fig. 5:**
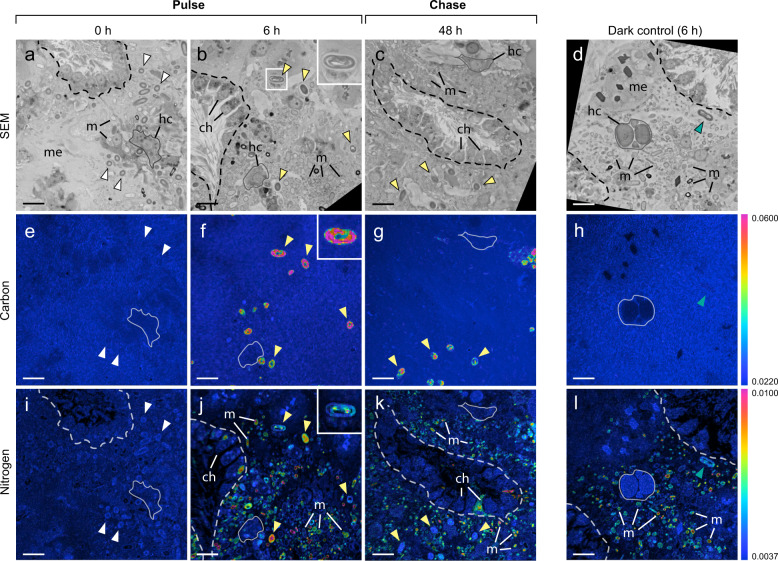
The inner sponge body of *Chondrilla caribensis*: single-cell visualization of inorganic ^13^C-bicarbonate and ^15^N-ammonium assimilation. SEM images (**a**–**d)** show the location of resident symbiotic cyanobacteria, microorganisms, and host cells. Corresponding NanoSIMS images show the distribution of ^13^C/^12^C (**e**–**h**) and ^15^N/^14^N (**i**–**l**) at the end of the pulse period (6 h) and chase period (48 h) in relation to non-labelled tissue (0 h) and the dark control. White arrows depict unlabelled cyanobacteria (0 h), yellow arrows depict cyanobacteria enriched in ^13^C and ^15^N, and the green arrow depicts a ^15^N-enriched cyanobacterium. See Fig. 4 for NanoSIMS scale. ch choanocyte, hc host cell, m symbiotic microorganism, me mesohyl. Scale bars are 5 µm. Dashed lines delineate choanocyte chambers. The white rectangle in (**b**) shows a close up of a cyanobacterium (inset).

**Fig. 6:**
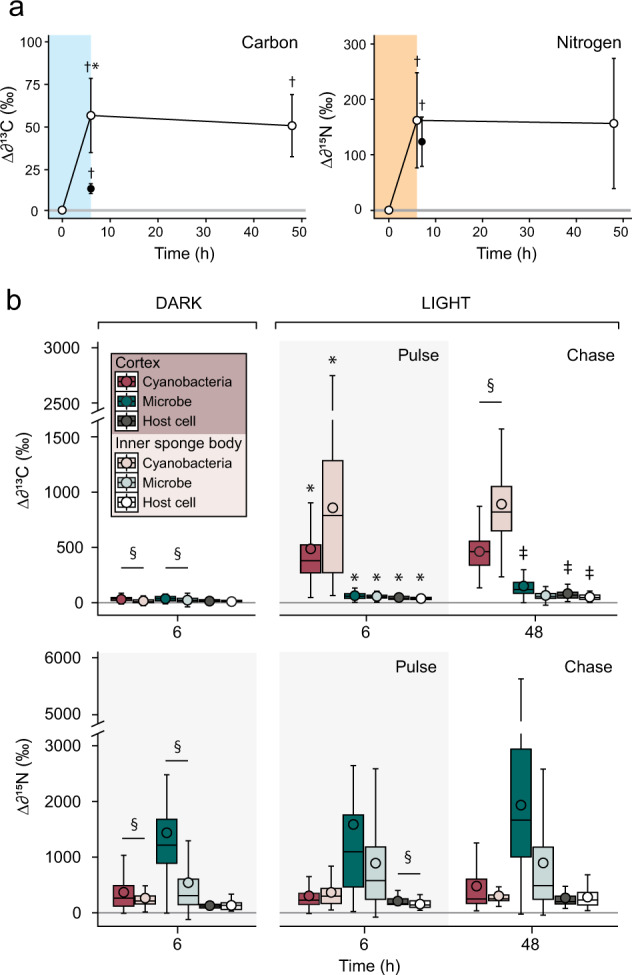
Assimilation of ^13^C-bicarbonate and ^15^N-ammonium at the holobiont- and single-cell-level in *Chondrilla caribensis*. **a** Above-background stable isotope enrichment of sponge tissue over the 48-h pulse-chase experiment. Data presented as mean ± SD (*n* = 4), coloured boxes represent the 6-h pulse period and filled circles represent the 6-h dark control. Significant differences (*p* < 0.05) are given for all time-points compared to *t* = 0 (†), and between *t* = 6 light and *t* = 6 dark (*; Supplementary Table 4). **b** Quantification of ^13^C- and ^15^N-assimilation in 3 regions of interest (ROI) within the sponge tissue: cyanobacteria, symbiotic microorganisms, and host cells, analyzed using NanoSIMS. Box plots display enrichment data (Δδ^13^C and Δδ^15^N) as quartiles (lower and upper hinges represent the 25th and 75th percentiles) and black circles represent mean values. Significant differences (*p* < 0.05) are given between *t* = 6 dark and *t* = 6 light (*), between *t* = 6 light (end pulse) and *t* = 48 (end chase) (‡), and between equivalent ROI of the cortex and inner sponge body (§) per time-point (Supplementary Table 5). The horizontal grey lines in **a**, **b** show the variation in unlabelled control sponges (as mean ± SD).

To visualize photosynthetic carbon fixation and trace its subsequent transfer, sponges were pulsed with ^13^C-bicarbonate and then moved to label-free seawater during the chase period. Correlating ultrastructural electron microscopy analysis with NanoSIMS revealed substantial carbon fixation by cyanobacteria at the end of the pulse period (Figs. 4 and 5), during which >99% of all cyanobacterial cells became enriched in ^13^C (Supplementary Fig. 3). Carbon fixation by these symbionts resulted in significant isotopic enrichment of the bulk sponge tissue after 6 h (Fig. 6a; Supplementary Table 4). While cyanobacteria of the inner sponge body were on average more highly enriched than those of the cortex (Fig. 6b), this pattern was not consistent across biological replicates. A portion of the remaining microbial community (i.e., all other prokaryotes) assimilated ^13^C during the pulse, with enrichment detected in 43 and 9% of cells in the cortex and inner sponge body, respectively. However, carbon fixation by symbiotic microorganisms was relatively trivial compared to that of cyanobacteria, with average δ^13^C-enrichment values being at least ten-fold lower (Fig. 6b). Host cells of the cortex and inner sponge body became significantly enriched in ^13^C during the pulse period (PERMANOVA pairwise tests *T*_0_ vs. *T*_6_, all *p*_(perm)_ < 0.01; Supplementary Table 5). This does not represent direct uptake, but rather translocation of organic carbon from cyanobacteria (and, to a lesser extent, other prokaryotic symbionts) to the host.

The isotopic enrichment of cyanobacteria did not change during the chase period, and cells remained highly enriched in ^13^C after 48 h (Fig. 6b). There was a concurrent increase in ^13^C assimilation by symbiotic microorganisms of the cortex and all host cells between 6 and 48 h (PERMANOVA pairwise tests *T*_6_ vs. *T*_48_, all *p*_(perm)_ < 0.05; Supplementary Table 5). This increase indicates further translocation from cyanobacteria to the host, as carbon assimilation was dominated by cyanobacteria during the 6-h pulse. Sponge cells containing engulfed, enriched cyanobacteria could be seen in the cortex (Fig. 4g, k), and of the 65 enriched host cells found, 14 contained visible intracellular ^13^C-labelled cyanobacteria. Due to the 2-dimensional nature of NanoSIMS images, some of these enriched host cells may have contained engulfed cyanobacteria that were not visible. However, the relatively low proportion (21%) of enriched cells containing cyanobacteria suggests that phagocytosis of cyanobacteria by host cells was not the only mode of translocation. Furthermore, only 1 out of 19 enriched host cells in the inner sponge body contained an intracellular cyanobacterium during the chase period.

There were low but significant levels of inorganic ^13^C-assimilation into the bulk tissue of *C. caribensis* during the dark control (Fig. 6a; Supplementary Table 4), but enrichment into specific cell types was not visually discernible at the single-cell level (Figs. 4h and 5h). Average ^13^C-enrichment values for all cell types did not significantly exceed background levels after 6 h (Supplementary Table 5), although a small portion of cyanobacteria and symbiotic microorganisms became enriched in ^13^C (2–24%, Supplementary Table 3). No host cells assimilated ^13^C during the dark control (0% of cells enriched), confirming that enrichment during the light pulse was driven by light-dependent carbon fixation by symbionts rather than uptake via anaplerotic pathways. Dark fixation rates of the holobiont were on average 4.4 times lower than light fixation rates (Fig. 6a).

### Ammonium assimilation is mediated by the broader sponge microbiome

Ammonium assimilation was driven by the entire microbial community of *C. caribensis* rather than cyanobacteria alone (Figs. 4 and 5). More than 90% of symbiotic microorganisms and cyanobacteria throughout the tissue became enriched in ^15^N within 6 h (Supplementary Fig. 3), with the average ^15^N-enrichment of symbiotic microorganisms at least a factor of 5 and 2 higher than that of cyanobacteria (Fig. 6b; Supplementary Table 3) in the cortex and inner sponge body, respectively. The majority of host cells (>97%) became enriched in ammonium during the 6-h pulse. While some of these cells contained hotspots of ^15^N-enrichment, indicative of engulfed microbial symbionts (Fig. 4k), many host cells were enriched without evidence of phagocytosis, indicating direct uptake or recycling of nitrogen produced during microbial ammonium assimilation. Choanocytes that had consumed ^15^N-enriched bacteria from the incubation medium may have also transferred metabolites to host cells located in the mesohyl via transcytosis. There were no significant differences between ^15^N-enrichment values for the light and dark pulse across all cell types (PERMANOVA pairwise tests *T*_6_ vs. *T*_6(d)_, all *p*_(perm)_ > 0.05; Supplementary Table 5), nor at the holobiont level (Fig. 6a), indicating that ammonium assimilation was not light dependent. During the chase period, ^15^N enrichment of symbiotic microorganisms and cyanobacteria of the cortex and all host cells increased (Fig. 6b), but these differences were not significant (PERMANOVA pairwise tests *T*_6_ vs. *T*_48_, all *p*_(perm)_ > 0.05; Supplementary Table 5). No clear patterns of translocation emerged during the chase period, although it is likely that high variability in ammonium assimilation at the holobiont level at 48 h (Fig. 6a) obscured any translocation dynamics.

## Discussion

Studies on the nutritional modes of sponges have predominantly quantified heterotrophy or autotrophy in isolation, hampering our understanding of their relative contribution to mixotrophy and how this strategy enables sponges to adapt to dynamic marine environments. Furthermore, our understanding of the role of photoautotrophy in sponges hosting photosymbionts is largely informed from species that are net phototrophic or live in high-light environments. Thus, a vast potential for photoautotrophy in net heterotrophic sponges that reside in low-light habitats and host photosymbionts has been overlooked. Here, we show that photoautotrophy supplements the diet of a sponge that dwells in lower light environments and can contribute significantly to the respiratory carbon requirements of the holobiont. Single-cell analysis confirmed that photosymbionts contribute to host metabolism, as photosynthetically fixed carbon was translocated from cyanobacteria to sponge cells, either via symbiont phagocytosis or the release and subsequent incorporation of photosynthates. We hypothesize that the contribution of photosynthetic carbon fixation to total sponge diet is underestimated in the many net heterotrophic sponges that host photosymbionts, and that mixotrophy is a widespread strategy for sponges residing in a range of illuminated shallow-water habitats, not solely restricted to high-light environments.

Despite the fact that *C. caribensis* predominantly resides in low-light reef environments, the transfer of fixed carbon from cyanobacteria to other members of the symbiosis can potentially offset the respiratory requirements of the holobiont by up to half (for established net phototrophic corals and sponges, this value can exceed 130% [57–59]). Moreover, photoautotrophic carbon acquisition actually exceeded the contribution of LPOC to the total mixotrophic carbon diet (7 vs. 1%, respectively). Such a contribution may help sponges buffer against nutritional stress induced by fluctuations in food availability by providing carbon necessary for metabolic maintenance. This may confer an important fitness advantage during periods of limited food supply. In warming seas, for example, enhanced water column stratification [60] can prolong periods of low food availability; such conditions have caused mass mortality events in filter-feeding communities in the Mediterranean [61]. While many studies have assessed the contribution of photoautotrophy to the respiratory demand of sponges [23–28], quantification of the respective contribution of photoautotrophy and heterotrophy to their total carbon budget has scarcely been studied. To our knowledge, this has only been measured in the shallow-water Symbiodiniaceae-hosting sponge *Cliona orientalis*, where photoautotrophic carbon acquisition was 10-fold higher than heterotrophic carbon acquisition [59]. Comparatively, heterotrophic carbon acquisition was 14-fold higher than photoautotrophic carbon acquisition for *C. caribensis*. The principally heterotrophic nature of this sponge supports the observation that mixotrophs favour one nutritional pathway over the other [62], and may be associated with trade-offs concomitant with maintaining cellular structures for both pathways [5]. Interestingly, *C. caribensis* resides across a spectrum of light environments, including very low-light cryptic reef habitats (<10 μmol photons m^−2^ s^−1^) and shallow reefs (<6 m water depth, >1000 μmol photons m^–2^ s^–1^) [42], demonstrating the adaptability of this species to a range of environmental conditions. It may be that shallow-reef sponges are more reliant on photoautotrophy than their low-light counterparts, as higher irradiances can increase rates of carbon fixation and transfer from symbiont to sponge host, and result in higher P:R ratios [23, 28]. However, as respiration can be enhanced by increased photosynthesis [25], this remains to be tested.

The balance of the daily respiratory requirements of *C. caribensis* was met (and exceeded) by heterotrophy, with organic carbon providing an estimated 93% of total daily carbon uptake, indicating that these sponges are not carbon limited. The excess carbon and nutrients (e.g., N, P, Fe) provided by the ingestion of organic material can support growth, reproduction, and cellular turnover. Dissolved organic carbon accounted for 98% of the natural diet, which is comparable to previously reported contributions in shallow-water and deep-sea species [51, 63, 64]. Feeding rates were variable for both dissolved and particulate organic carbon, with some replicates showing no net uptake of either bacteria, phytoplankton, or DOC (Supplementary Table 6). Although analytical measurements may have missed very small changes in organic carbon concentrations, feeding rates can be affected by a multitude of factors, including the quantity and quality of ambient organic carbon [65], pumping rates [7], and the physiological status of the host. Dissolved organic carbon fluxes in particular exhibit high intraspecific variability, with some individuals being a sink for DOC and others a source during the day [64–66]. Uptake rates of DOC were higher in the dark compared to the light, and no net uptake or release of DOC was observed in the majority of replicates under light conditions. Day–night DOC uptake rates in sponges are largely unknown and may vary for species that host photoautotrophic symbionts. Similar to our findings, the only study to date that has measured day–night rates found elevated DOC uptake rates at night compared to the day in a photosymbiotic sponge [67]. Limited DOC uptake during the day in *C. caribensis* may have resulted from DOC release stimulated by the photosynthetic activity of symbiotic cyanobacteria, or diel variability in heterotrophic feeding, as some sponges exhibit plasticity in their feeding modes and can enhance heterotrophy in response to shading [19, 28] and increased turbidity [68].

In photoautotrophic symbioses, the translocation of organic carbon from symbiont to heterotrophic host occurs primarily via the release and uptake of small organic molecules and/or the digestion of symbionts [69–71]. Our findings indicate that both translocation pathways may occur in *C. caribensis*, confirming that this sponge benefits from its photosynthetic symbionts. Phagocytosis of cyanobacterial cells was observed in NanoSIMS (red arrows in Fig. 4) and TEM images (Supplementary Fig. 4), with some of these symbiont cells undergoing apparent intracellular digestion. However, many sponge cells assimilated ^13^C throughout the pulse-chase without apparent engulfment of cyanobacteria, indicating that the release of organic carbon by cyanobacteria and direct uptake by host cells is also a likely pathway of translocation in this symbiosis. This would be consistent with early work by Wilkinson [72], who found that symbiotic cyanobacteria release glycerol and an organic phosphate, which can be readily metabolized by host cells or function as reducing equivalents [19], as well as previous studies suggesting that cyanobacteria digestion represents a minor translocation route in sponges [73, 74]. It is thought that cyanobacteria avoid digestion by host cells by (i) omitting the biosynthesis of an antigen which triggers recognition and phagocytosis by sponge amoebocyte cells [75], and (ii) expression of eukaryotic-like proteins, which are believed to modulate eukaryotic–prokaryotic interactions and enable symbiont persistence within the host [76–78]. Therefore, the low occurrence of symbiont digestion observed herein could represent elimination of compromised cyanobacteria [79].

No loss of ^13^C-enrichment by cyanobacteria was detected between 6 and 48 h, indicating that much of the photosynthetically fixed carbon was retained and the portion translocated to the host was relatively small. Low rates of photosynthate translocation have been described in a sponge–rhodophyte symbiosis, where it was posited that the extracellular nature of the association diminished the rate of nutrient transfer compared to intracellular symbioses [80]. Low photosynthetic rates by *C. caribensis* residing in low-light habitats may also limit the release and subsequent host utilization of “excess” photosynthates. For example, comparatively low abundances of glycogen, the dominant photosynthetic storage compound in cyanobacteria, were found in *Ca*. S. spongiarum cells of a low-irradiance sponge compared to a high-irradiance species, indicating lower photosynthetic activity and reduced nutritional benefit for the host [35]. In shallow-water Symbiodiniaceae symbioses, a large proportion of photosynthates (<85%) can be rapidly transferred to the host [57, 81, 82]. Furthermore, carbon fixed during the day by Symbiodiniaceae is rapidly lost during the night in corals [71]. Our data suggest that translocation may be reduced or occur over a longer time frame in *C. caribensis* compared to associations with Symbiodiniaceae dinoflagellates.

Inorganic carbon fixation within the sponge holobiont was further enhanced by the prokaryotic community of *C. caribensis*, exclusive of cyanobacteria. Carbon assimilation by symbiotic microorganisms during the 6-h light pulse may indicate internal recycling of carbon fixed by cyanobacteria or direct carbon fixation via photoautotrophy. Several symbionts of the family Rhodobacteraceae (Alphaproteobacteria) are capable of fixing carbon via anoxygenic photosynthesis [83], and members of this family are present in *C. caribensis*. Chemoautotrophic carbon fixation pathways are also associated with members of the *C. caribensis* microbiome. The reductive tricarboxylic acid cycle in Nitrospirae [84, 85] and the 3-hydroxypropionate/4-hydroxybutyrate (HP/HB) cycle in Thaumarchaeota [85, 86] fix inorganic carbon, and could explain ^13^C-assimilation by a small portion of the prokaryotic community during the dark control. Nearly all symbionts became enriched in ^15^N during the 6-h pulse, demonstrating the ubiquity of ammonium fixation pathways across diverse microbial taxa in *C. caribensis*. Furthermore, NanoSIMS images (Figs. 4 and 5) qualitatively showed that ammonium assimilation appeared to be largely a microbially mediated process in this sponge. Several symbionts enriched in ^15^N were further observed to be phagocytosed by sponge cells (Fig. 4k), demonstrating that a portion of microbially assimilated nitrogen was also translocated to the host.

## Conclusions

Here, we quantified the mixotrophic diet of a sponge residing at 20 m water depth and demonstrate that even at low light levels, photoautotrophy can supplement holobiont nutrition via the provision of symbiont-derived organic carbon. We highlight the importance of quantifying the contribution of photoautotrophy to holobiont metabolism in sponge species that do not show net primary productivity (i.e., P:R ratios <1) and propose a more comprehensive approach to sponge nutrition, encompassing both autotrophy and heterotrophy, to better understand sponges’ trophic plasticity. Our understanding of the extent of sponge mixotrophy is currently hampered by a lack of physiological data. In the Caribbean, for example, over a third of sponges host photosymbionts [18, 87], but directly linking this to the trophic nature of the holobiont requires additional photosynthesis/respiration data that are available for only a limited number of species [23, 27, 28]. Future research should focus on assessing the prevalence and magnitude of mixotrophy in sponges from a range of environments, as this nutritional mode can have profound influences on biogeochemical cycling and food-web structure [5]. Investigating how sponges adjust heterotrophy and autotrophy in response to varying environmental conditions is also needed, as the relative contribution of these feeding modes to host metabolism can be affected by parameters such as irradiance, temperature, and food availability [23, 65, 88]. Given that sponges are a significant component of the benthic biomass of many shallow-water communities worldwide [7], and that the occurrence of photosymbiont-bearing sponges can be high (e.g., 30–72% [18, 89, 90]), the contribution of sponge photoautotrophy to ecosystem productivity is an important metric that remains to be resolved.

## Supplementary information

Supplementary Material

Data Set 1

## Data Availability

All data generated or analyzed during this study are included in this published article and its supplementary information files.
