# Supplementary material for: Harnessing solar power: photoautotrophy supplements the diet of a low-light dwelling sponge
Source: ISME J. 2022 Jun 2;16(9):2076–86. doi: 10.1038/s41396-022-01254-3 (PMC9381825; doi:10.1038/s41396-022-01254-3)
Supplement: Supplementary file 1 — Supplementary Material [file 41396_2022_1254_MOESM1_ESM.pdf]

Supplementary Information

Harnessing solar power: photoautotrophy supplements the diet of a low-light dwelling sponge

Authors: Meggie Hudspith, Jasper M. de Goeij, Mischa Streekstra, Niklas A. Kornder, Jeremy Bougoure, Paul Guagliardo, Sara Campana, Nicole N. van der Wel, Gerard Muyzer, Laura Rix.

Contents

Supplementary Methods 2
Supplementary Figures 7
- Supplementary Fig. 1 7
- Supplementary Fig. 2 8
- Supplementary Fig. 3 9
- Supplementary Fig. 4 10

Supplementary Tables 11
- Supplementary Table 1 11
- Supplementary Table 2 11
- Supplementary Table 3 12
- Supplementary Table 4 14
- Supplementary Table 5 14
- Supplementary Table 6 16

References 16

Supplementary Methods

Microbial community analysis

Sponge tissue samples taken from *Chondrilla caribensis* specimens collected *in situ* (20 m water depth, $n = 3$) were assessed for microbial community analysis using Illumina sequencing of the 16S rRNA gene. DNA was extracted from tissue samples using the DNeasy Blood and Tissue Kit (Qiagen, Germany). Frozen tissue was first homogenized using Powerbead tubes provided in the PowerSoil DNA isolation kit (MoBio): the pre-loaded solution of the tubes was removed and filled with 180 µL of ATL buffer and 20 µL of proteinase K, and approximately 0.5 cm^2 of finely minced frozen tissue was added and the tubes vortexed horizontally for 15 min. Samples were incubated overnight (56 °C) then centrifuged at 6000 x *g* for 2 min or until all un-lysed tissue was pelleted. The supernatant was transferred onto the spin column and extraction proceeded as per the manufacturer's protocol. The DNA concentration was measured with a Qubit dsDNA BR Assay Kit and Qubit Fluorometer (Invitrogen, CA, USA). DNA was stored at -20 °C until further analysis. 16S rRNA gene amplicon sequencing was performed using the two-stage protocol published by Naqib *et al.* [1]. Briefly, the 16S rRNA gene was amplified using the primer pair CS1 515F-CS2 926R. Subsequently, 1 µl of PCR product was amplified in a second PCR to attach the unique barcode. Amplicon libraries were sequenced on an Illumina MiSeq platform (MiSeq FGx; Illumina, San Diego, USA) at the DNA Service Facility, University of Illinois, Chicago, USA. Sequences were analyzed with the software program QIIME2, using the DADA2 algorithm and the Silva taxonomy database. MicrobiomeAnalyst was used to create the taxonomic abundance profiles [2].

Photosynthesis–irradiance incubations

Sponge individuals were transferred to the indoor wet-lab facility of CARMABI 24 h before use. Individuals were then transferred to 2-L chambers, which were partially submerged in an outdoor running-seawater aquarium (100 L, flow rate of 3 L min^{-1}) to maintain ambient temperatures. Chamber lids were equipped with a magnetic stirring device and an optical probe and temperature sensor were inserted through airtight ports in the lid for continuous dissolved oxygen (DO) measurements (OXY-4 mini, PreSens, Germany) and temperature compensation, respectively. Photosynthetically active radiation (PAR; 400–700 nm wavelength) was measured using an Odyssey integrating light data recorder (Dataflow Systems Ltd, Christchurch, New Zealand) which had been calibrated using a LI-192 underwater quantum sensor (LI-COR Biosciences, USA). Incubations were performed on cloudless afternoons to ensure constant irradiance levels and were run concurrently with seawater only controls.

Sponges were acclimatized to dark conditions for 10 min prior to measuring dark respiration. For each irradiance level, oxygen fluxes were calculated using the linear slope of oxygen concentration (recorded every 10 s), excluding a 1 min transitory period between light intervals, and corrected for the seawater controls.

Light model parameterization

Variation in daily surface irradiance was modelled [3] using:

$$I_{st} = I_{\max} \times sine\left(\frac{t \times \pi}{P}\right) \times (1 - SR) \quad (1)$$

where I_{st} is the surface light intensity at time t (μmol photons m^{-2} s^{-1}), I_{max} is the maximum light intensity (1760 μmol photons m^{-2} s^{-1}) at solar noon, t is the number of hours since dawn, P is the number of hours of daylight (12 h), and SR is the fraction of incident light reflected at the surface (15 %) [4]. Variation in light at 20 m depth was then derived using:

$$I_{dt} = I_{st} \times e^{(-K \times D)} \quad (2)$$

as per [3], where I_{dt} is the irradiance at a given depth (D, 20 m), I_{st} is derived from Eq. 1, and K is the absorption coefficient (0.09 m^{-1}). An irradiance profile was modelled as irradiances are not affected by day-to-day fluctuations in light intensity [5]. To construct a typical light regime experienced by *C. caribensis* residing on vertical surfaces at 20 m depth, irradiances calculated in Eq. 2 were parameterized against light data collected at the CARMABI house reef (Supplementary Table 1). A correction factor of 0.202 was applied to I_{dt} to generate a light profile reflecting the upper range of irradiances experienced by *C. caribensis* at 20 m on vertical surfaces (receiving mostly indirect sunlight), with a midday maximum of 50 μmol photons m^{-2} s^{-1}.

Natural diet incubations: water sample processing and analysis

For the natural diet incubations, each chamber lid was fitted with a magnetic stirring device, and a HOBO dissolved oxygen logger (Onset Computer Corporation, MA, USA) was inserted through a sample port, which logged DO and temperature every minute. The temperature during *in situ* incubations was 28.6 ± 0.1°C (mean ± SD). Water samples taken during *in situ* natural diet incubations were stored at 4 °C at the surface and processed within 5 h of sampling. Samples for bacterial (1 mL) and phytoplankton (3.5 mL) abundances were transferred to cryovials pre-loaded with glutaraldehyde (2 % (v/v) final concentration) and a formaldehyde + hexamine solution (0.5 % (v/v) formaldehyde and 0.3 % (w/v) hexamine, final concentration), respectively. Samples were incubated for 1 h in the dark, flash frozen in liquid nitrogen and stored at -80 °C until analysis. Inorganic nutrient samples (8 mL) were filtered over 0.2 μm syringe filters (Thermo Fisher Scientific, MA, USA), collected in HDPE vials and stored at -20 °C. Samples for dissolved organic carbon (DOC) and total dissolved nitrogen (TDN) (20 mL) were filtered using clean in-line filter holders containing pre-combusted (450 °C, 4 h) GF/F filters (0.7 μm pore size, 25 mm, Whatman) into pre-combusted 20 mL EPA vials. Vials were acidified with 6–7 drops of HCl (37 %) and stored in the dark at 4 °C. In-line filter holders were pre-cleaned by successive flushing with 20 mL of 0.4 M HCl, Milli-Q water, and sample water.

Samples were transported to the Netherlands and analyzed at the Analytical Laboratory of the Institute for Biodiversity and Ecosystem Dynamics, University of Amsterdam. Phytoplankton and heterotrophic bacteria (high- and low-nucleic acid bacteria) were enumerated using a CytoFLEX flow cytometer (Beckman Coulter, USA). Cells were excited with a blue laser (488 nm) and detected on the basis of cell forward scatter and side scatter, green fluorescence (DNA stained cells), red fluorescence (chlorophyll) and orange fluorescence (phycoerythrin). Phytoplankton samples (50 μL) were analyzed at medium flow rate (30 μL min^{-1}). *Synechococcus* sp. (*Syne*) were identified by orange fluorescence emission, and *Prochlorococcus* sp. (*Pro)* by low red and orange fluorescence. Bacterial samples were stained with SYBR Green I nucleic acid gel stain (Molecular Probes, Inc) as previously

described [6] and 50 μL of sample was analyzed at medium flow rate. Flow cytometry data were analyzed using the software CytExpert v. 2 (Beckman Coulter, USA). Carbon and nitrogen contents of different cell populations were estimated using published conversion factors: *Syne,* 470 fg C per cell [7] and 50 fg N per cell [8]; *Pro,* 53 fg C per cell [9] and 9.4 fg N per cell [8]; heterotrophic bacteria, 20 fg C per cell and 5.4 fg N per cell [10]. Concentrations of dissolved inorganic nitrogen (DIN; NO_x [NO_2^- + NO_3^-] [11], NO_2^- and NH_4^+ [12]) were measured using a San^{++} continuous flow analyzer (Skalar). Dissolved organic carbon and total dissolved nitrogen (TDN) concentrations were measured by high-temperature combustion using a total organic carbon analyzer (Shimadzu) as per Campana *et al.* [13]. Dissolved organic nitrogen (DON) concentrations were calculated by subtracting DIN from TDN, and net DON uptake rates are presented in Supplementary Figure 2 and Supplementary Table 6.

Initial dissolved organic matter (DOM) removal rates were estimated using a bi-exponential 2G model [14, 15]. This simplified model describes the uptake of DOM (as DOC or DON) over time, assuming that this complex pool of compounds is composed of two major fractions: a refractory component with a slow turnover rate (C_s) and a labile component with a fast turnover rate (C_f). In a well-mixed system, these components will be consumed according to their specific removal rate constants, K_s for the refractory component and K_f for the labile component. Total DOM removal rates can then be expressed as the sum of the individual uptake rates of both fractions:

$$\frac{dDOC/DON}{dt} = -(K_s C_s + K_f C_f) \tag{3}$$

The concentration of DOM is then expressed as a function of time, *t,* where:

$$DOC/DON(t) = C_{s,0} \times e^{-kst} + C_{f,0} \times e^{-kft} \tag{4}$$

The model is then fit to the experimental data by estimating the model variables using a minimalization routine with 10 000 iterations. Estimates of the model variables (based on the integration of the model over the entire time frame of the incubation) are then used to calculate initial removal rates of DOM, based on the tangent of the model at t_0, and is given by:

$$Flux_{DOC/DON} = -(K_s C_{s,0} + K_f C_{f,0}) \tag{5}$$

Initial net uptake rates of LPOC and LPON were estimated assuming the exponential clearance of planktonic cells in a well-mixed system [16]. The planktonic community was characterized by heterotrophic bacteria, *Synechococcus,* and *Prochlorococcus,* and uptake rates were calculated from the tangent of the exponential model at t_0. Seawater control data (pooled per light condition) for both DOM and POM were fitted using linear regression. Where fitted models were not significant, and *post hoc* linear regression did not show significant removal or release rates, uptake rates were set to 0.

Pulse-chase experimental conditions

Sponges were transferred from storage cages at the house reef to the wet-lab facilities of CARMABI between 12 and 24 h prior to incubation and maintained in 100-L flow-through aquaria, supplied with water pumped from the adjacent reef at 10 m water depth (3 L min^{-1}). Individuals were transferred to 3-L airtight incubation chambers filled with isotopically labelled ASW and the lids closed ensuring no headspace. The ammonium concentration supplied (5 µM) is higher than ambient concentrations on shallow Curaçaon reefs (0.3–1.7 µM NH_4^+; this study and [17, 18]) but is lower than local elevated concentrations caused by nutrient runoff (~ 7 µM NH_4^+ [19]). Chambers were partially submerged in an outdoor flow-through aquaria to ensure near *in situ* temperature. The average temperature across all *ex situ* incubations was 28.9 ± 0.4 °C. Incubation medium (labelled ASW) was refreshed after 3 h by removing approximately 2300 mL and replenishing with fresh incubation medium. This was done to remove any accumulated metabolites, ensure sufficient oxygenation, and maximize isotopic labelling. Chamber lids were equipped with a magnetic stirring device and temperature and oxygen probes, as for the photosynthesis–irradiance incubations. Dissolved oxygen concentrations decreased linearly during incubations, demonstrating active pumping of sponge individuals, and did not fall below 10 % of starting concentrations. At the end of the 6-h pulse, sponges were rinsed in label-free natural seawater and transferred to flow-through aquaria for the 42-h chase. At each sampling time-point (t = 0, 6, 6 (dark), 48 h), sponges were removed from incubation chambers or the flow-through aquaria, rinsed in 0.7 µm filtered seawater and Milli-Q, and imaged for planar surface area before tissue samples were taken. Throughout the pulse-chase experiment, light was reduced using shade cloth to reflect light levels experienced by *C. caribensis* at 20 m water depth (noon irradiance ~ 50 µmol photons m^{-2} s^{-1}, measured using an Odyssey PAR logger). The incubations for sponges sampled at 6 and 48 h were conducted 2 days apart due to logistical restrictions, but measured light levels were similar on these days (25 ± 19 and 27 ± 20 µmol photons m^{-2} s^{-1} for $t = 6$ and $t = 48$ sponges, respectively).

Elemental and stable isotope analysis

To measure the carbon (C) and nitrogen (N) content and stable isotopic ratios of sponge tissue, samples were measured using a Vario Isotope Tube Elemental Analyser (EA, Elementar GmbH, Germany) coupled with a BioVision IRMS (Elementar UK Ltd). Frozen samples were freeze-dried, weighed, and homogenized using a ball mill (Retsch, Germany). Samples were directly weighed into tin boats ($\delta^{15}N$) and silver boats ($\delta^{13}C$), and samples for $\delta^{13}C$ were then acidified overnight using 4 M HCl to remove inorganic C. Isotopic ratios are expressed in standard delta notation relative to the international standards Vienna Pee Dee Belemnite and atmospheric nitrogen:

$$\delta^{13}C \text{ or } \delta^{15}N = \left(\frac{R_{sample}}{R_{ref}} - 1\right) \text{ x } 1000 \quad (6)$$

Where R_{sample} and R_{ref} represent the ratio of $^{13}C/^{12}C$ and $^{15}N/^{14}N$ in the sample or standards.

Microscopy images

To visualize symbiotic cyanobacteria in the tissue of *C. caribensis* and characterize their zonation, fluorescence microscopy and transmission electron microscope (TEM) imaging was

performed on fixed samples of sponge tissue. For fluorescence microscopy, samples were fixed in 2.5 % glutaraldehyde + 1 % paraformaldehyde in 0.1 M Sorensen's buffer (pH 7.4) and then cryo-protected by infiltration with 2.3 M sucrose in 0.1 M Sorensen's buffer. Samples were sectioned (200 nm) using a cryo ultramicrotome (Ultracut UC6/FC6, Leica Microsystems, Austria) at École Polytechnique Fédérale de Lausanne (EPFL), Switzerland, and imaged using a Zeiss LSM 700 inverted confocal microscope (Zeiss, Germany). For TEM analysis, samples were fixed and embedded as per Hudspith *et al.* [20] and then 100 nm sections mounted on TEM grids. Sections were stained with uranyl acetate and lead citrate and then imaged with a FEI Tecnai T12 TEM (120 kv) at the Electron Microscopy Centre Amsterdam (EMCA).

The width of cyanobacterial cells in the outer cortex and inner sponge body was measured from TEM images using ImageJ software (v. 1.53). Only cells showing a clear center and peripheral thylakoids were measured. A total of 98 cells were measured in the cortex and 40 in the inner sponge body, from 2 sponge individuals.

Supplementary Figures

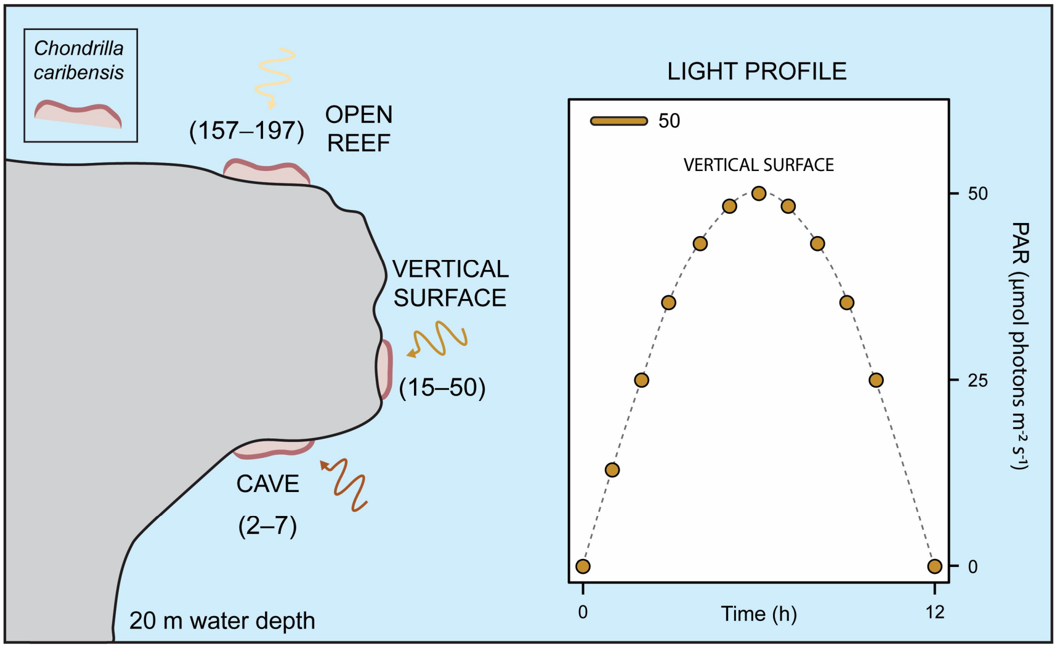

Supplementary Fig. 1 – Schematic diagram showing a simplified characterization of light regimes typically experienced by *C. caribensis* at 20 m water depth. This sponge can be found from 15 to 25 m on exposed benthic surfaces, vertical surfaces and cryptic reef habitats, such as caves or underhangs, with greater abundances at 20 m water depth. The numbers in parentheses represent the range in peak irradiances received in these environments at midday (see Supplementary Table 1), measured in μmol photons m^{-2} s^{-1}. A daily light profile was modelled based on maximal irradiances received on vertical surfaces at 20 m, which was integrated against *P–E* curves to calculate daily gross primary productivity. PAR; photosynthetically active radiation (400–700 nm).

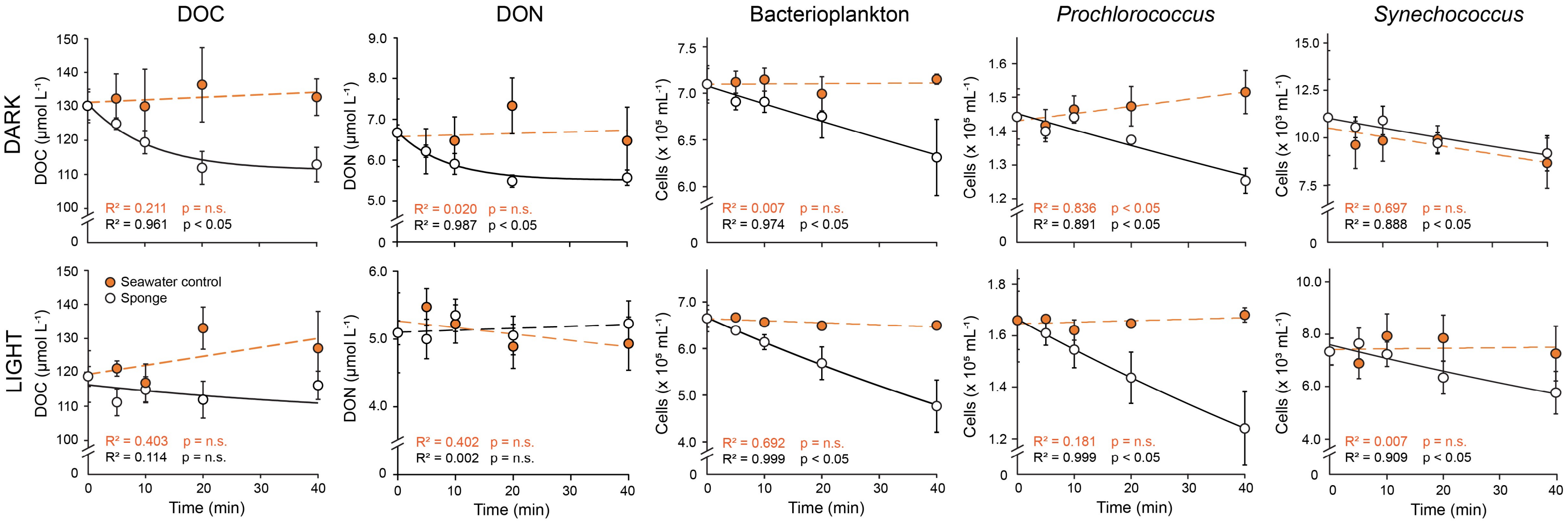

Supplementary Fig. 2 – Uptake of DOC, DON, bacterio- and phytoplankton by *C. caribensis* during natural diet incubations under dark and light conditions. Data are displayed as mean ± standard error (*n* = 6 for sponge incubations [white circles], *n* = 3 for seawater controls [orange circles], per light condition) standardized to average initial concentrations. Sponge data were fitted with a bi-exponential 2G (DOC/N) or exponential model (bacterio- and phytoplankton), and seawater controls with a linear model. Models presented here are for visualization purposes only; individual models were run for each replicate on raw (unstandardized) data. For individual uptake rates per replicate see Supplementary Table 6. DOC, dissolved organic carbon; DON, dissolved organic nitrogen.

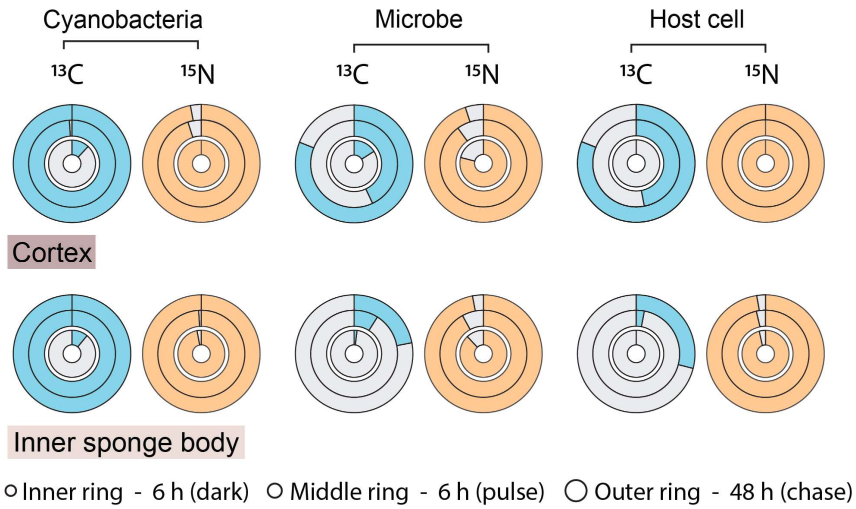

Supplementary Fig. 3 - The proportion of cells enriched in ^{13}C and ^{15}N for three regions of interest (ROI) within the tissue of *C. caribensis* during the pulse-chase experiment. Enrichment of cyanobacteria, symbiotic microorganisms, and host cells, in the cortex and inner sponge body, were measured using NanoSIMS. Shown are distributions of enriched (coloured) and non-enriched (grey) cells. ROI were deemed enriched if isotopic enrichment exceeded three times the standard deviation of unlabelled control ROI. For details (including *n* per ROI, per time-point) see Supplementary Table 3.

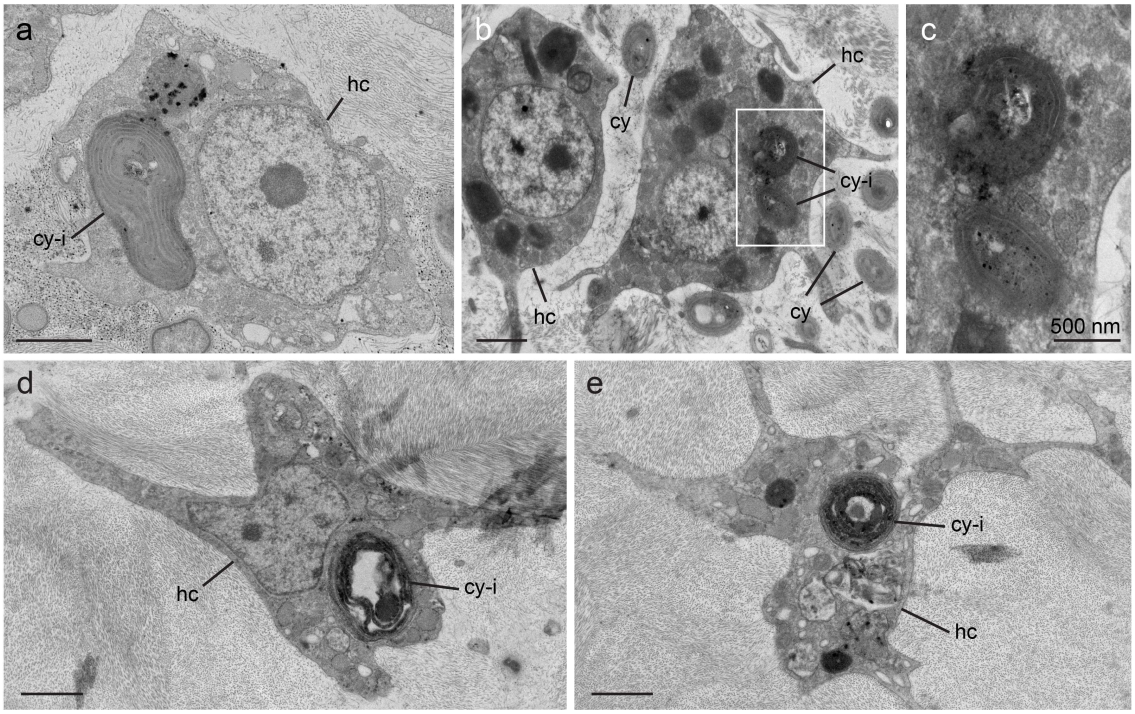

Supplementary Fig. 4 – Interactions between cyanobacteria and host cells in the inner sponge body (**a**) and cortex (**b–e**) of *C. caribensis*. Transmission electron microscope images (**a–e**) show cyanobacteria closely associated and engulfed by amoebocyte-like host cells. Several intracellular cyanobacteria appear to be undergoing digestion (**b, d**). cy, cyanobacteria; cy-i, intracellular cyanobacteria; hc, host cell. **c** is an enlargement of the white rectangle in **b**. Scale bars are 1 µm unless stated otherwise.

Supplementary Tables

Supplementary Table 1 – Typical maximal light levels experienced by *C. caribensis* at the house reef and within the wet-lab facilities of CARMABI. For the house reef, data were collected between midday and 1 pm on three consecutive days in December 2019 using an Odyssey PAR logger. Measurements were taken from 20 m water depth at multiple positions on the reef where *C. caribensis* resides to assess the light environments experienced by this sponge. A midday maximum of 50 PAR was chosen to parameterize the light model, representing the upper range of light levels experienced by *C. caribensis* residing on vertical surfaces at 20 m water depth. Light measurements (midday maximum) were also taken from the cages where collected sponges were stored (house reef), and the indoor holding aquaria prior to the pulse-chase incubations and *P–E* curves. PAR units = µmol photons m^{-2} s^{-1}.

Description	PAR	Chosen PAR for model
Upward-facing exposed surfaces (= open reef irradiances at 20 m)	157–197	
Vertical, sideward-facing surfaces	15–50	50
Cave or underneath a boulder/overhang	2–7	
Cages at 15 m water depth	< 30	
Holding flow-through aquaria	< 28	

Supplementary Table 2 - Photokinetic parameters derived from the *P–E* curves of *C. caribensis* replicates (r; $n = 4$). P_{max}, maximum gross photosynthetic rate; $P_{m.n}$, maximum net photosynthetic rate; E_k, saturating irradiance; E_c, compensation irradiance; α, initial slope of *P–E* curve; R_d, dark respiration (µmol O_2 cm^{-2}_{sponge} h^{-1}). The fit of the model parameters (P_{max} and E_k) to the *P–E* data are shown; values in bold are statistically significant ($p < 0.05$).

Photokinetic parameters								P_{max}			E_k		
r	P_{max}	$P_{m.n}$	E_k	E_c	α	R_d	R^2	SE	t	*p*	SE	t	*p*
1	1.218	0.750	96.0	46.5	0.01	-0.468	0.907	0.16	7.64	**0.0006**	35.6	2.70	**0.043**
2	1.779	0.543	28.5	33.8	0.06	-1.236	0.992	0.04	50.4	**<0.0001**	2.34	12.2	**<0.0001**
3	1.637	0.337	57.2	90.4	0.03	-1.300	0.936	0.13	13.0	**<0.0001**	15.5	3.70	**0.014**
4	1.695	0.636	46.3	45.4	0.04	-1.059	0.940	0.11	15.5	**<0.0001**	12.0	3.85	**0.012**

Supplementary Table 3 - Summary of the isotopic enrichment of different regions of interest (ROI) within the cyanobacteria-bearing sponge *C. caribensis* after a pulse of ^{13}C-bicarbonate and ^{15}N-ammonium. Shown are average $\Delta\delta^{13}C$ and $\Delta\delta^{15}N$ (in ‰) values for 3 ROI in the cortex and inner sponge body (IS) for pooled replicates and each individual replicate, extracted from NanoSIMS data. The proportion of enriched cells (Enr.) as a percentage of the total is given; cells are deemed enriched if their δ-values exceed 3 x the standard deviation (SD) of equivalent non-labelled ROIs. *n* = number of analyzed cells per ROI category. 6 (d) represents the 6 h dark control.

			Carbon ($\Delta\delta^{13}C$)								Nitrogen ($\Delta\delta^{15}N$)						
Time (h)	ROI	*n*	Mean	SD	SE	Enr. (%)	*n*	Mean	SD	SE	Mean	SD	SE	Enr. (%)	Mean	SD	SE
0	Microbial symbiont	25	0	14.9	3.0						0	51.4	10.3				
	Microbial symbiont (IS)	1373	0	22.4	0.6						0	37.1	1.0				
	Cyanobacteria	142	0	12.6	1.1						0	26.0	2.2				
	Cyanobacteria (IS)	103	0	14.5	1.4						0	19.2	1.9				
	Host cell	24	0	6.1	1.2						0	7.3	1.5				
	Host cell (IS)	48	0	15.8	2.3						0	17.1	2.5				
6 (d)	Microbial symbiont	79	28.3	31.0	3.5	24	19	14.8	25.9	6.0	1774.7	1263.9	142.2	95	908.8	1090.1	250.1
							60	32.6	31.5	4.1					2048.9	1196.4	154.5
	Microbial symbiont (IS)	2595	1.5	36.9	0.7	2	812	13.8	33.6	1.2	669.5	927.6	18.2	88	405.0	422.1	14.8
							1783	-4.0	37.0	0.9					790.0	1060.7	25.1
	Cyanobacteria	372	25.6	20.6	1.1	24	76	9.0	29.5	3.4	452.0	404.1	21.0	91	1068.4	406.8	46.7
							296	29.8	14.9	0.9					293.8	200.7	11.7
	Cyanobacteria (IS)	62	-6.1	34.7	4.4	11	15	39.1	28.0	7.2	317.5	238.3	30.3	97	619.6	281.2	72.6
							47	-20.5	21.9	3.2					221.1	109.6	16.0
	Host cell	15	-7.3	20.8	5.4	0	15	-7.3	20.8	5.4	157.4	93.3	24.1	100	157.4	93.3	24.1
	Host cell (IS)	41	-11.7	29.2	4.6	0	21	-1.4	14.5	3.2	161.0	100.6	15.7	93	97.9	52.3	11.4
							20	-22.5	36.5	8.2					227.3	96.8	21.7
6	Microbial symbiont	60	42.9	35.7	4.6	43	19	61.4	40.2	9.2	1592.0	1734.2	223.9	90	1667.6	2057.6	472.0
							41	34.3	30.3	4.7					1556.9	1589.6	248.3
	Microbial symbiont (IS)	2143	36.7	30.0	0.6	9	1235	35.0	28.1	0.8	892.7	976.9	21.1	92	972.0	1024.8	29.2

							908	38.9	32.2	1.1					784.8	897.0	29.8
	Cyanobacteria	174	475.8	387.7	29.4	99	154	389.6	214.6	17.3	302.8	410.9	31.2	95	261.1	392.6	31.6
							20	1139.4	688.7	154.0					623.9	416.9	93.2
	Cyanobacteria (IS)	90	854.7	705.4	74.4	100	42	283.5	193.9	29.9	364.9	272.0	28.7	99	239.8	186.6	28.8
							48	1354.5	602.3	86.9					474.5	289.0	41.7
	Host cell	17	26.3	29.5	7.2	47	7	43.1	38.0	14.4	208.1	92.8	22.5	100	152.8	20.7	7.8
							10	14.5	14.5	4.6					246.9	104.8	33.2
	Host cell (IS)	39	16.6	15.1	2.4	3	14	13.8	7.7	2.1	153.7	77.2	12.4	97	127.7	83.3	22.3
							25	18.1	17.9	3.6					168.3	71.2	14.2
48	Microbial symbiont	64	132.8	129.7	16.2	81	27	117.2	42.8	8.2	1941.0	1308.3	163.5	95	2311.3	1116.4	214.9
							17	20.9	32.6	7.9					884.5	1092.5	265.0
							20	249.1	163.1	36.5					2339.1	1266.9	283.3
	Microbial symbiont (IS)	1697	46.1	52.8	1.3	22	535	74.6	54.8	2.4	897.1	1061.0	25.8	97	1419.0	1097.6	47.5
							355	32.5	38.3	2.0					1350.6	1459.4	77.5
							807	33.2	49.5	1.7					351.6	256.7	9.0
	Cyanobacteria	75	451.8	181.8	21.0	100	48	427.3	139.3	20.1	478.1	666.5	77.0	97	199.1	104.6	15.1
							3	211.4	58.1	33.5					1767.4	676.7	390.7
							24	530.8	225.9	46.1					874.9	907.5	185.2
	Cyanobacteria (IS)	38	889.4	484.9	78.7	100	5	417.2	217.1	97.1	302.4	178.0	28.9	100	174.1	44.5	19.9
							2	627.5	261.3	184.8					180.9	48.0	34.0
							31	982.4	480.4	86.3					331.0	184.8	33.2
	Host cell	80	62.8	71.2	8.0	81	27	83.1	45.5	8.8	265.1	198.2	22.2	100	181.3	57.3	11.0
							30	15.6	18.2	3.3					254.4	111.5	20.4
							23	100.5	101.5	21.1					377.6	314.3	65.6
	Host cell (IS)	65	30.7	27.8	3.5	29	27	50.6	23.8	4.6	280.2	198.6	24.6	98	353.7	242.1	46.6
							19	18.1	21.8	5.0					291.7	174.8	40.1
							19	15.2	21.1	4.8					164.2	50.7	11.6

Supplementary Table 4 – Results of one-factor permutational analysis of variance (PERMANOVAs) testing the effect of treatment time-point (0, 6 (dark), 6, 48 h) on differences in carbon (C; $\delta^{13}C$) and nitrogen (N; $\delta^{15}N$) enrichment of bulk tissue of *C. caribensis*. Ti, time point; d.f., degrees of freedom; SS, sum of squares; MS, mean sum of squares; $p_{(perm)}$, permutational *p* value; d, 6 h dark control; $p_{(MC)}$, Monte Carlo *p* values. Values in bold are statistically significant ($p < 0.05$).

	Main test						Pairwise tests				
								C		N	
	Source	df	SS	MS	Pseudo-*F*	$p_{(perm)}$	Groups	t	$p_{(MC)}$	t	$p_{(MC)}$
C	Ti	3	14924	4974.7	10.145	**0.0012**	0 vs d	8.657	**0.0003**	5.277	**0.0024**
	Res	12	5884.4	490.37			0 vs 6h	5.884	**0.0015**	3.776	**0.0096**
	Total	15	20809				0 vs 48h	3.811	**0.0071**	2.268	0.0649
N	Ti	3	93631	31210	2.9399	0.0563	d vs 6h	4.224	**0.0064**	0.562	0.5886
	Res	12	127400	10616			d vs 48h	2.996	**0.0248**	0.752	0.4853
	Total	15	221030				6h vs 48h	0.751	0.4792	0.425	0.6883

Supplementary Table 5 - Results of individual two-factor PERMANOVAs testing for differences in ^{13}C- or ^{15}N-enrichment between treatment time-points (0, 6 (dark), 6, 48 h) and each ROI category in the sponge *C. caribensis.* ROI were scanned in both the cortex and inner sponge body (IS). Ti, time point; RO, ROI; d.f., degrees of freedom; SS, sum of squares; MS, mean sum of squares; $p_{(perm)}$, permutational *p* value. Values in bold are statistically significant ($p_{(perm)} < 0.05$). Cy, cyanobacteria; d, 6 h dark control; Hc, host cell; M, symbiotic microorganism.

Main Test					
**Carbon**					
Source	df	SS	MS	Pseudo-F	$p_{(perm)}$
Ti	3	2.15E+07	7.17E+06	714.32	**0.0002**
RO	5	6.36E+07	1.27E+07	1266.9	**0.0002**
TixRO	15	7.06E+07	4.71E+06	469.15	**0.0002**
Res	9397	9.43E+07	10038		
Total	9420	2.35E+08			
**Nitrogen**					
Source	df	SS	MS	Pseudo-F	$p_{(perm)}$
Ti	3	9.56E+07	3.19E+07	44.428	**0.0002**
RO	5	2.33E+08	4.67E+07	65.097	**0.0002**
TixRO	15	8.90E+07	5.93E+06	8.2773	**0.0002**
Res	9397	6.74E+09	7.17E+05		
Total	9420	8.05E+09			

Pairwise tests					
		Carbon		Nitrogen	
Level	Groups	t	$p_{(perm)}$	t	$p_{(perm)}$

M	0 vs d	4.3909	n.s	6.9956	**0.0002**
	0 vs 6h	5.7817	**0.0002**	4.5731	**0.0002**
	0 vs 48h	5.0894	**0.0002**	7.3898	**0.0002**
	d vs 6h	3.1628	**0.0013**	0.71879	0.478
	d vs 48h	3.9255	**0.0002**	0.77012	0.4492
	6h vs 48h	5.1898	**0.0002**	1.2703	0.214
Cy	0 vs d	3.1355	n.s	31.241	n.s.
	0 vs 6h	14.611	**0.0002**	8.7635	n.s.
	0 vs 48h	29.541	**0.0002**	8.5528	n.s.
	d vs 6h	10.469	**0.0002**	13.592	n.s.
	d vs 48h	20.957	**0.0002**	0.44915	0.6582
	6h vs 48h	0.51178	0.6134	2.5309	n.s.
Hc	0 vs d	1.6144	0.0998	8.288	**0.0002**
	0 vs 6h	4.2595	**0.0002**	10.993	**0.0002**
	0 vs 48h	4.2989	**0.0002**	6.5286	**0.0002**
	d vs 6h	3.6674	**0.0002**	1.5394	0.1316
	d vs 48h	3.764	**0.0008**	2.0557	**0.0398**
	6h vs 48h	2.0694	**0.0402**	1.1549	0.2514
M (IS)	0 vs d	1.4147	0.1594	26.732	**0.0002**
	0 vs 6h	38.918	**0.0002**	33.843	**0.0002**
	0 vs 48h	30.183	**0.0002**	31.312	**0.0002**
	d vs 6h	35.488	**0.0002**	8.0454	n.s.
	d vs 48h	32.506	**0.0002**	7.4187	n.s.
	6h vs 48h	6.9484	n.s.	0.13387	0.8992
Cy (IS)	0 vs d	1.5608	0.1206	13.476	**0.0002**
	0 vs 6h	12.298	**0.0002**	13.582	**0.0002**
	0 vs 48h	18.707	**0.0002**	17.081	**0.0002**
	d vs 6h	9.5901	**0.0002**	1.1096	0.271
	d vs 48h	14.526	**0.0002**	0.33696	0.745
	6h vs 48h	0.2764	0.7812	1.3021	0.2034
Hc (IS)	0 vs d	2.3997	**0.0116**	10.922	**0.0002**
	0 vs 6h	4.966	**0.0002**	13.413	**0.0002**
	0 vs 48h	6.8712	**0.0002**	9.7373	**0.0002**
	d vs 6h	5.4027	**0.0002**	0.36198	0.7258
	d vs 48h	7.5033	**0.0002**	3.5613	n.s.
	6h vs 48h	2.9292	**0.0038**	3.8022	n.s.
0 h	M vs M (IS)	1.87E-07	1.000	Negative	
	Cy vs Cy (IS)	8.81E-08	1.000	4.20E-08	1.000
	Hc vs Hc (IS)	2.27E-08	1.000	1.71E-08	1.000
d	M vs M (IS)	6.3833	**0.0002**	10.304	**0.0002**
	Cy vs Cy (IS)	9.9749	**0.0002**	2.546	**0.0112**
	Hc vs Hc (IS)	0.53991	0.6222	0.12172	0.9006
6 h	M vs M (IS)	1.5734	0.1048	5.4583	n.s.
	Cy vs Cy (IS)	5.5923	n.s.	1.5814	0.2102
	Hc vs Hc (IS)	1.634	0.1016	1.9632	**0.0264**
48 h	M vs M (IS)	11.87	n.s.	7.81	n.s.
	Cy vs Cy (IS)	6.9355	**0.0002**	1.4719	0.1014
	Hc vs Hc (IS)	3.4185	n.s.	0.69958	0.657

*For those groups where both location (PERMANOVA) and dispersion (PERMDISP) were significantly different, ordination plots (non-metric multi-dimensional scaling) were examined. Where there was no clear separation of groups on nMDS plots, $p_{(perm)}$ values were deemed not significant (n.s.).

Supplementary Table 6 – Organic carbon and nitrogen uptake rates of *C. caribensis,* measured from *in situ* natural diet incubations and corrected for seawater controls. Exponential clearance and 2G models were fitted to bacterio/phytoplankton and DOC/N data, respectively. r, replicate; Bact*,* bacterioplankton; *Pro*, *Prochlorococcus*; *Syne*, *Synechococcus*; POC/N, particulate organic carbon/nitrogen; DOC/N, dissolved organic carbon/nitrogen.

		μmol C h^{-1}				μmol C cm^{-2} h^{-1}		μmol C g DW^{-1} h^{-1}	
Light condition	r	Bact	*Pro*	*Syne*	POC	POC	DOC	POC	DOC
Dark	5	0.13	0	0	0.13	0.01	16.01	0.31	742.3
	6	0.40	0	0	0.40	0.02	15.77	1.18	757.6
	7	1.63	0.49	0.04	2.17	0.10	2.98	4.91	142.8
	8	0	0.15	0	0.15	0.01	7.65	0.62	399.6
	9	0	0.25	0	0.25	0.02	13.22	0.87	588.6
	10	0.31	0.37	0	0.67	0.04	0	1.28	0
Light	5	2.03	1.28	0.51	3.82	0.19	0	8.92	0
	6	2.58	1.47	0.82	4.87	0.30	0	14.55	0
	7	1.68	0.85	0	2.53	0.12	0	5.73	0
	8	0.25	0	0	0.25	0.02	0	1.01	0
	9	0.81	0.26	0	1.07	0.08	0	3.65	0
	10	0.70	0	0	0.70	0.04	8.02	1.33	260.6
		μmol N h^{-1}				μmol N cm^{-2} h^{-1}		μmol N g DW^{-1} h^{-1}	
Light condition	r	Bact	*Pro*	*Syne*	PON	PON	DON	PON	DON
Dark	5	0.09	0	0	0.092	0.005	0	0.274	0
	6	0.03	0	0	0.031	0.002	0	0.072	0
	7	0.38	0.07	0.08	0.531	0.025	1.17	1.202	56.24
	8	0	0.02	0	0.023	0.002	1.40	0.094	73.37
	9	0	0.04	0	0.039	0.003	0.62	0.132	27.67
	10	0.07	0.06	0	0.126	0.007	0	0.241	0
Light	5	0.47	0.19	0.05	0.710	0.044	0	1.660	0
	6	0.60	0.22	0.08	0.895	0.045	0	2.673	0
	7	0.39	0.13	0	0.519	0.024	0	1.174	0
	8	0.06	0	0	0.057	0.004	0	0.233	0
	9	0.19	0.04	0	0.226	0.017	0	0.776	0
	10	0.16	0	0	0.161	0.009	1.19	0.308	38.706

References

1. Naqib A, Poggi S, Wang W, Hyde M, Kunstman K, Green SJ. Making and sequencing heavily multiplexed, high-throughput 16S ribosomal RNA gene amplicon libraries using a flexible, two-stage PCR protocol. *Gene expression analysis*. 2018. Humana Press, New York, NY, pp 149–169.
2. Dhariwal A, Chong J, Habib S, King IL, Agellon LB, Xia J. MicrobiomeAnalyst: a web-based tool for comprehensive statistical, visual and meta-analysis of microbiome data. *Nucleic Acids Res* 2017; **45**: W180–W188.
3. Cheshire AC, Wilkinson CR. Modelling the photosynthetic production by sponges on Davies Reef, Great Barrier Reef. *Mar Biol* 1991; **109**: 13–18.

4. Larkum A, Barrett J. Light-harvesting processes in algae. *Advances in botanical research*. 1983. Academic Press, pp 1–219.
5. Cheshire AC, Wilkinson CR, Seddon S, Westphalen G. Bathymetric and seasonal changes in photosynthesis and respiration of the phototrophic sponge *Phyllospongia lamellosa* in comparison with respiration by the heterotrophic sponge *Ianthella basta* on Davies Reef, Great Barrier Reef. *Mar Freshw Res* 1997; **48**: 589–599.
6. Marie D, Partensky F, Jacquet S, Vaulot D. Enumeration and cell cycle analysis of natural populations of marine picoplankton by flow cytometry using the nucleic acid stain SYBR Green I. *Appl Environ Microbiol* 1997; **63**: 186–193.
7. Verity PG, Robertson CY, Tronzo CR, Andrews MG, Nelson JR, Sieracki ME. Relationships between cell volume and the carbon and nitrogen content of marine photosynthetic nanoplankton. *Limnol Oceanogr* 1992; **37**: 1434–1446.
8. Bertilsson S, Berglund O, Karl DM, Chisholm SW. Elemental composition of marine *Prochlorococcus* and *Synechococcus*: Implications for the ecological stoichiometry of the sea. *Limnol Oceanogr* 2003; **48**: 1721–1731.
9. Campbell L, Nolla HA, Vaulot D. The importance of *Prochlorococcus* to community structure in the central North Pacific Ocean. *Limnol Oceanogr* 1994; **39**: 954–961.
10. Lee S, Fuhrman JA. Relationships between biovolume and biomass of naturally derived marine bacterioplankton. *Appl Environ Microbiol* 1987; **53**: 1298–1303.
11. Grasshoff K, Kremling K, Ehrhardt M. Methods of Seawater Analysis, 3rd Edition. 2009. John Wiley & Sons.
12. Helder W, de Vries RT. An automatic phenol-hypochlorite method for the determination of ammonia in sea- and brackish waters. *Netherlands J Sea Res* 1979; **13**: 154–160.
13. Campana S, Hudspith M, Lankes D, Kluijver A De, Demey C, Schoorl J, et al. Processing of naturally sourced macroalgal- and coral-dissolved organic matter (DOM) by high and low microbial abundance encrusting sponges. 2021; **8**: 1–13.
14. de Goeij JM, van Duyl FC. Coral cavities are sinks of dissolved organic carbon (DOC). *Limnol Oceanogr* 2007; **52**: 2608–2617.
15. de Goeij JM, van den Berg H, van Oostveen MM, Epping EHG, van Duyl FC. Major bulk dissolved organic carbon (DOC) removal by encrusting coral reef cavity sponges. *Mar Ecol Prog Ser* 2008; **357**: 139–151.
16. Scheffers SR, Nieuwland G, Bak RPM, Van Duyl FC. Removal of bacteria and nutrient dynamics within the coral reef framework of Curaçao (Netherlands Antilles). *Coral Reefs* 2004; **23**: 413–422.
17. Gast GJ, Wiegman S, Wieringa E, Van Duyl FC, Bak RPM. Bacteria in coral reef water types: removal of cells, stimulation of growth and mineralization. *Mar Ecol Prog Ser* 1998; **167**: 37–45.
18. Alexander BE, Mueller B, Vermeij MJA, van der Geest HHG, de Goeij JM. Biofouling of inlet pipes affects water quality in running seawater aquaria and compromises sponge cell proliferation. *PeerJ* 2015; **2015**: 1–15.
19. den Haan J, Huisman J, Brocke HJ, Goehlich H, Latijnhouwers KRW, Van Heeringen S, et al. Nitrogen and phosphorus uptake rates of different species from a coral reef community after a nutrient pulse. *Sci Rep* 2016; **6**: 1–13.
20. Hudspith M, Rix L, Achlatis M, Bougoure J, Guagliardo P, Clode P., et al. Subcellular view of host–microbiome nutrient exchange in sponges: insights into the ecological success of an early metazoan–microbe symbiosis. *Microbiome* 2021; **9**: 1–15.
